# *Xanthomonas citri* jumbo phage XacN1 exhibits a wide host range and high complement of tRNA genes

**DOI:** 10.1038/s41598-018-22239-3

**Published:** 2018-03-14

**Authors:** Genki Yoshikawa, Ahmed Askora, Romain Blanc-Mathieu, Takeru Kawasaki, Yanze Li, Miyako Nakano, Hiroyuki Ogata, Takashi Yamada

**Affiliations:** 1 Institute for Chemical Research, Kyoto University, Gokasho, Uji 611-0011 Japan; 2 Department of Molecular Biotechnology, Graduate School of Advanced Sciences of Matter, Hiroshima University, Higashi-Hiroshima, 739-8530 Japan; 3 Department of Microbiology, Faculty of Science, Zagazig University, 44519 Zagazig, Egypt; 4 Hiroshima Study Center, The Open University of Japan, Hiroshima, 730-0053 Japan

## Abstract

*Xanthomonas* virus (phage) XacN1 is a novel jumbo myovirus infecting *Xanthomonas citri*, the causative agent of Asian citrus canker. Its linear 384,670 bp double-stranded DNA genome encodes 592 proteins and presents the longest (66 kbp) direct terminal repeats (DTRs) among sequenced viral genomes. The DTRs harbor 56 tRNA genes, which correspond to all 20 amino acids and represent the largest number of tRNA genes reported in a viral genome. Codon usage analysis revealed a propensity for the phage encoded tRNAs to target codons that are highly used by the phage but less frequently by its host. The existence of these tRNA genes and seven additional translation-related genes as well as a chaperonin gene found in the XacN1 genome suggests a relative independence of phage replication on host molecular machinery, leading to a prediction of a wide host range for this jumbo phage. We confirmed the prediction by showing a wider host range of XacN1 than other *X. citri* phages in an infection test against a panel of host strains. Phylogenetic analyses revealed a clade of phages composed of XacN1 and ten other jumbo phages, indicating an evolutionary stable large genome size for this group of phages.

## Introduction

Tailed bacteriophages (phages) with genomes larger than 200 kbp are commonly named “jumbo phages”^1^. They usually show large virions, being capable of enclosing a larger genome than those of other smaller phages^2^. Jumbo phages are classified into several evolutionarily unrelated clades^3,4^. The largest reported phage is *Bacillus* phage G with a 160 nm capsid and a 453 nm tail. Phage G genome assembly is 497,513 bp long (accession number: NC_023719). Recently, a new group of jumbo phages has been recognized^5^. This group, named Rak2-like phages, is composed of phages with a genome longer than 300 kbp including *Klebsiella* phage vB_KleM-RaK2^6^, *Escherichia* phage PBECO 4^7^, *Cronobacter* phage vB_CsaM_GAP32^5^, *Escherichia* phage 121Q (accession number: NC_025447), *Klebsiella* phage K64-1^8^, *Enterobacteria* phage vB_PcaM_CBB^9^, and *Serratia* phage BF^10^. Rak2-like phages encode genes homologous to the “core genes” that are conserved among T4-related phages^6,11^. However, given the level of sequence divergence, the lack of many of the core genes of T4-related phages, and a large number of genes that are shared within Rak2-like phages but not with T4-like phages, Rak2-like phages were proposed to be only distantly related to T4-like phages^9^.

*Xanthomonas citri* (Gammaproteobacteria) is the causative agent of Asian citrus canker, one of the most serious plant diseases, which can lead to significant economic losses worldwide^12,13^. *X citri* is a rod-shaped Gram-negative bacterium with polar flagella. The first *X. citri* genome was determined for the strain 306, and consists of a 5.18 Mbp chromosome (64.8% G + C) and two plasmids, pXAC33 (33.7 kbp, 60.8% G + C) and pXAC64 (64.9 kbp, 61.4% G + C)^14^. In total, 4,429 protein-encoding genes and 62 RNA genes were identified in the genome. Currently genome sequences are available for 40X*. citri* strains. Many *X. citri* genes were suggested to be involved in the pathogenicity and virulence^15^. *X. citri* forms a biofilm to attach its host. The biofilm results from the production of extracellular polysaccharide (xanthan) and ensures the virulence and epiphytic survival of *X. citri* cells prior to the development of citrus canker^16^. To control citrus canker disease, several integrated management strategies have been proposed including exclusion, eradication, and sanitation^17,18^, but this disease continues to be a serious problem for field-grown crops in many countries because of the limited effectiveness of the current methods^19^. Phages are currently seen as an alternative to agrichemicals such as cooper sprays and antibiotics to control bacterial diseases^19,20^. Especially, jumbo phages are expected to be useful because of their generally wide host ranges and sustainable infection strategies^21^.

Recently, thanks to progress of standard screening methods, an increasing number of jumbo phages have been isolated from various bacteria^2^. In this study, we report the genome sequence of a newly isolated *Xanthomonas* jumbo virus (phage) XacN1.

## Results and Discussion

### Isolation and initial characterization of XacN1

**Figure 1:**
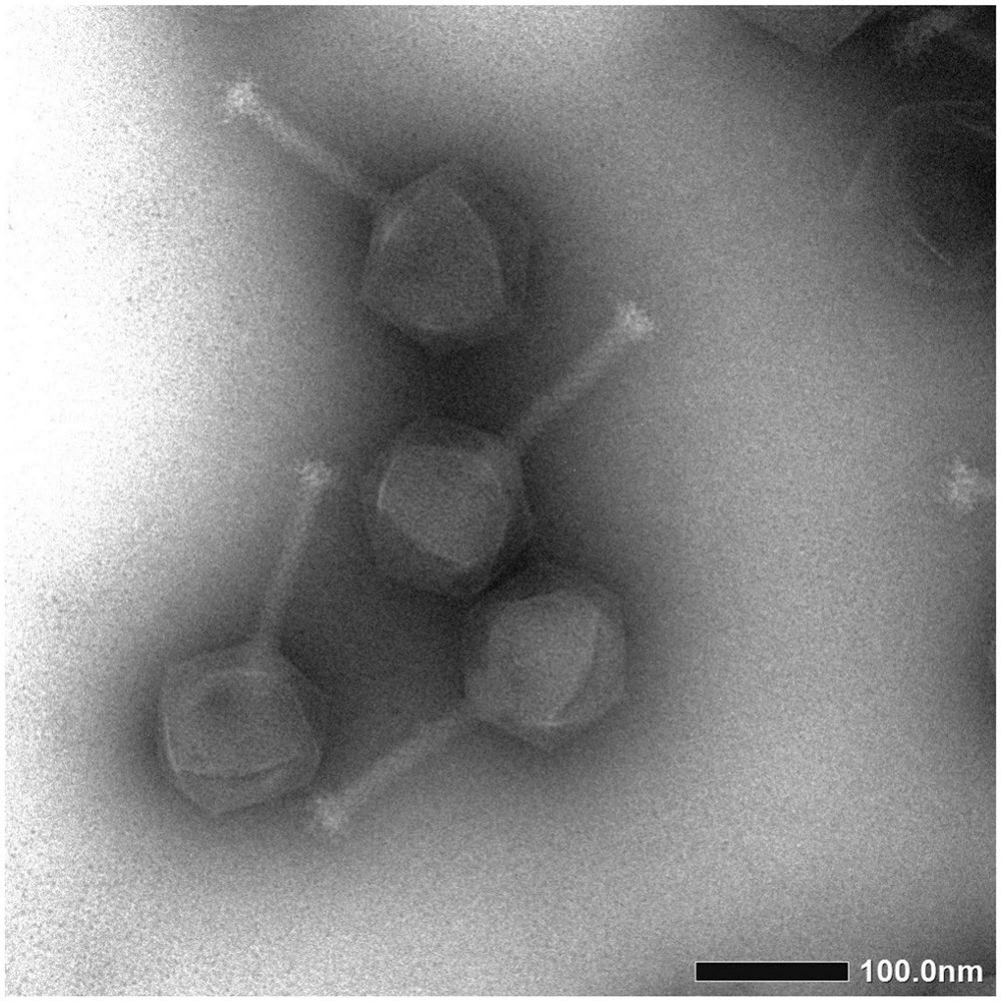
Electron micrograph of XacN1. Phage particles were stained with 1% ammonium molybdate. Bar = 100 nm.

XacN1 was isolated from a soil sample collected from orange groves in Akitsu, Japan, by plaque assay with *X. citri* strain MAFF 301080 as the host. The phage formed clear plaques (1–2 mm) on 0.3% top agar, but formed very small plaques (<0.5 mm) when the top agar concentration was increased to 0.45% (Supplementary Figure S1). Morphological features of XacN1 particles revealed by electron microscopy are characteristic to myoviruses (Fig. 1).

### The genome of XacN1 harbors long direct terminal repeats

In pulsed-field gel electrophoresis (PFGE) analyses, the genomic DNA of XacN1 gave a band at approximately 390 kbp (Supplementary Fig. S2). The sequenced XacN1 genome was a linear double-stranded DNA of 384,670 bp with a 50% G + C content, representing the fourth largest among sequenced phage genomes. Three larger phage genomes are those of *Bacillus* phage G (497,513 bp), *Agrobacterium* phage Atu_ph07 (490,380 bp, accession number: MF403008), and *Salicola* phage SCTP-2 (440,001 bp, accession number: MF360958). The XacN1 genome was predicted to contain 592 putative open reading frames (ORFs) and 58 tRNA-like sequences (Supplementary Table S2). Among the 592 ORFs, 223 (38%) showed significant sequence similarities to known sequences, and putative functions were assigned to 124 ORFs (21%). Only one ORF (ORF328; glycosyltransferase) showed its best BLAST hit to proteins encoded in *Xanthomonas*, suggesting a limited level of horizontal gene transfer between XacN1 and its host.

The extremities of the genome sequence showed 65,875 bp direct terminal repeats (DTRs; position 1 to 65,875 and 318,796 to 384,670). Each of these repeated regions encoded 131 ORFs and 29 tRNA-like sequences (including one tRNA pseudogene). The XacN1 DTR unit (66 kbp) is nearly three times longer than that of *Enterobacteria* phage vB_PcaM_CBB (22,456 bp)^9^, the longest previously recognized DTR in phage genomes. The sequenced phage G genome is 498 kbp, while it was previously estimated to be 670 kbp by PFGE^22,23^. There has been no report providing evidence of redundancy in the phage G genome. If phage G genome possesses unassembled DTRs as in our preliminary analysis of the XacN1 genome (see Methods), the length of phage G DTR unit is likely to be 170 kbp long. It is known that the terminal repeat regions of *Enterobacteria* phage T7 DNA molecules are essential for T7 phage production^24^. DTRs were also found in the genome of *Bacillus* phage CampHawk (13,772 bp)^25^ and *Bacillus* phage SPO1 (13,185 bp)^26^. In SPO1, DTRs encode proteins necessary for the subversion of the biosynthetic machinery of the host, which occurs at an early stage of phage infection^27^. The presence of tRNA genes in the XacN1 DTRs suggests that these genomic regions encode functions to take over host molecular machinery during infection. ORFs in these regions contained lytic enzyme genes such as a cell wall hydrolase gene and a M23 family peptidase gene (Supplementary Table S2). The M23 peptidase domain has been observed in endolysins of phages, including phages infecting *Thermus*, *Lactococcus*, *Entercococcus*, *Rhodococcus*, *Clostridium*, and *Lactobacillus*^28^. We could not identify other notable functions in the DTR regions.

### Functional content suggests a relatively independent phage replication and translation

#### tRNA genes and tRNA processing genes

**Figure 2:**
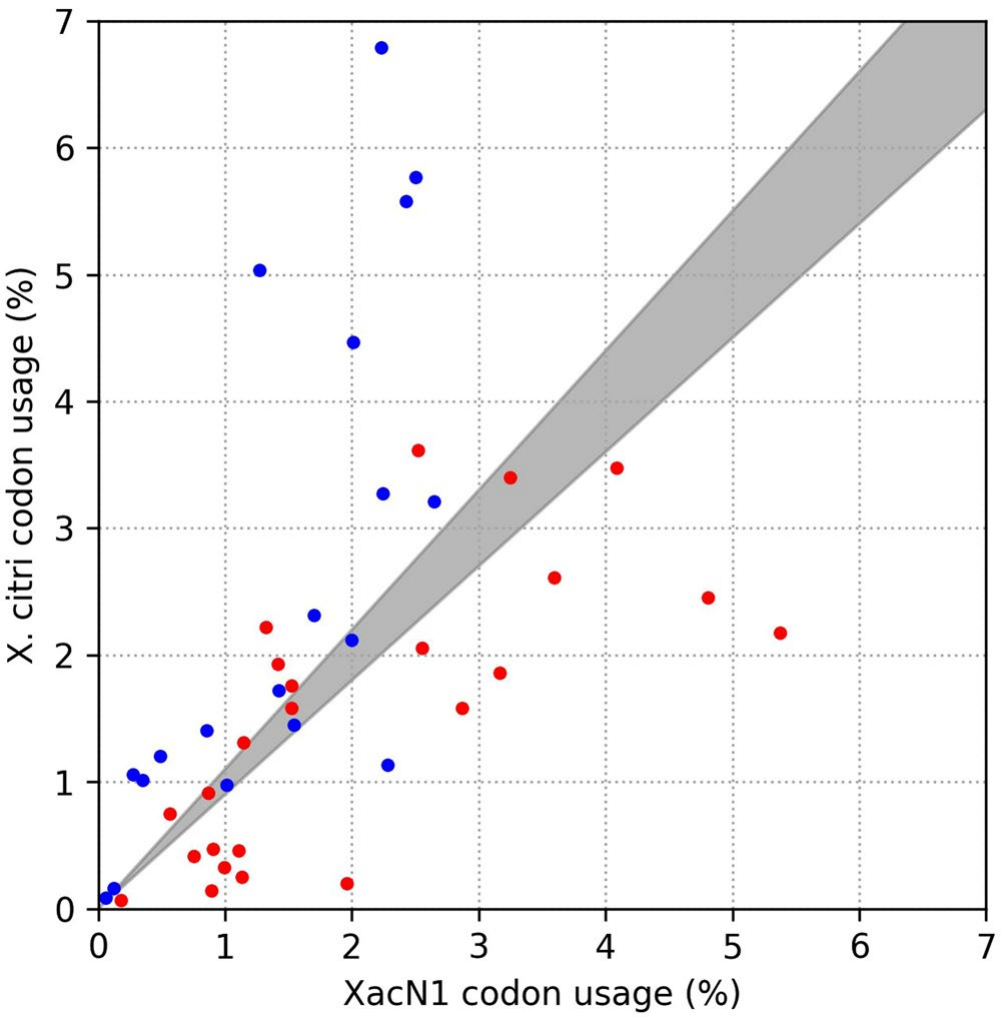
Comparison of codon usages between XacN1 and *X. citri*. Red dots represent codons for which corresponding tRNAs are encoded in both XacN1 and *X. citri*. Blue dots represent codons for which corresponding tRNAs are encoded only in *X. citri*. In gray area, relative codon frequencies are similar between XacN1 and *X. citri* (i.e. ratio of relative codon frequencies is between 0.9 and 1.1).

Of the 29 tRNA-like sequences in each of the DTRs, 26 encoded canonical tRNAs that potentially carry amino acids (Supplementary Table S3). These canonical tRNA genes corresponded to all the twenty amino acids. The remaining sequences were two suppressor tRNA genes (for read-through of stops) and a pseudogene. The anticodon of the suppressor tRNA genes were CUA, suggesting read-through of UAG (amber) stop codon. *In silico* read-through of amber stop codons (N = 18) in the XacN1 genome, however, did not uncover any additional sequence similarities against databases after the amber stop codons. Therefore, we could not identify potential genes targeted by these suppressor tRNA genes. All the XacN1 tRNA species (or “types” based on anticodons) were found encoded in the host genome except for the suppressor tRNAs. Therefore, the numerous XacN1 tRNA genes do not increase anticodon variety (i.e. decoding capacity) available for phage gene translation. However, a comparison of codon usages between XacN1 and its host revealed a significant tendency that the phage tRNA species correspond to codons that are more highly used in the phage than in the host genome (Fisher’s exact test: *p* = 0.00012) (Fig. 2), suggesting that the phage modulates the concentrations of tRNA species by encoding tRNA genes and adapts translation processes to its own codon usages^29^. Although tRNA molecules are generally stable, some bacteria are known to show a fast turnover of tRNAs. For example, the average half-life of tRNA is 11.8 minutes in *Vibrio cholerae*^30^. Such a fast turnover is thought to be carried out through a specific tRNA degradation pathway^31^. XacN1 completes the latent phase of its life cycle within 90 minutes and the growth cycle takes 240 minutes with a burst size of about 30 pfu per cell (Supplementary Fig. S3). Therefore, host tRNA molecules may become deficient during XacN1 infection. The phage encoded tRNAs may enable an efficient translation of phage genes in such a cellular condition. It is also known that some phages of the *Myoviridae* family encoding tRNA genes have a wide host range^32^. Therefore, the large number of tRNA genes of XacN1 also suggests a wide host range. Indeed, XacN1 showed a wider host range than siphovirus phage Cp1 and podovirus phage Cp2^33^, neither of which encodes tRNA genes, by infecting nine of ten tested *X. citri* strains isolated in Japan (Supplementary Table S1).

In addition to tRNA genes, XacN1 encodes seven ORFs that are likely involved in tRNA processing. First, four ORFs are directly related to tRNA maturation: tRNA^His^ guanylyltransferase (ORF126 and ORF585), glutamyl-tRNA amidotransferase (ORF360), and CCA tRNA nucleotidyltransferase (ORF446). Second, XacN1 encodes polynucleotide kinase-phosphatase (PNKP; ORF197) and SAM-dependent methyltransferase (ORF198), which is homologous to 3′ terminal RNA ribose 2′-O-methyltransferase Hen1. It is known that two bacterial proteins PNKP and Hen1 form a stable complex that repairs tRNAs^34^. Finally, XacN1 encodes a peptidyl-tRNA hydrolase (ORF295), which decreases the accumulation of peptidyl-tRNAs generated during the initiation, elongation and termination steps of protein biosynthesis. Including putatively functional 56 tRNA genes, XacN1 thus encodes 63 translation related genes.

#### Chaperonin

A chaperonin-like protein encoded by ORF282 showed sequence similarity (43.1% amino acid sequence identity) to the co-chaperone GroES of *Enterorhabdus caecimuris* (NCBI Reference Sequence: WP_016309324.1) and may function for chaperonin-assisted folding of polypeptides. No detectable viral homologs for the XacN1 ORF282 was found in the databases. During phage replication, chaperonin is usually supplied from the host and involved in the generation of phage particles^35^. However, recent metagenomic data indicated that chaperonin-encoding viruses are more common than previously thought and geographically widespread in marine ecosystems^36,37^. By encoding its own chaperonin, XacN1 virion assembly process might not be totally dependent on the host chaperonin machinery.

#### Glycosyltransferases

XacN1 encodes two glycosyltransferases (ORF328 and ORF334). Typically, viral proteins are glycosylated by host-encoded glycosyltransferases and the glycan portion of viral glycoproteins is consequently host-specific. However, glycosylation of the *Paramecium bursaria* Chlorella virus 1 (PBCV-1) major capsid protein, Vp54, is at least partially performed by virus-encoded glycosyltransferases^38^. Glycosylation of structural proteins has rarely been identified in phages. Some phage-encoded glucosyltransferases are known to modify viral DNA to protect it from host restriction endonucleases^39^. In XacN1, at least two glycoproteins were detected by proteomic analysis (Supplementary Fig. S4). Therefore, glycoproteins of XacN1 may be generated by the phage encoded glycosyltransferases as in the case of PBCV-1.

#### DNA replication and nucleotide metabolism

XacN1 encodes a large number of enzymes involved in DNA replication, synthesis and modification (Supplementary Table S5). All of these genes except for DNA polymerase III epsilon subunit (ORF223) showed homologs in other viruses. These genes are conserved in T4-like phages^11^. Like other phages^40^, XacN1 also encodes enzymes involved in nucleotide metabolism (Supplementary Table S5), including CMP/dCMP deaminase (ORF93, ORF204, ORF552), thymidylate synthase/dihydrofolate reductase (ORF186).

### Specialized infection-related protein encoding genes

**Figure 3:**
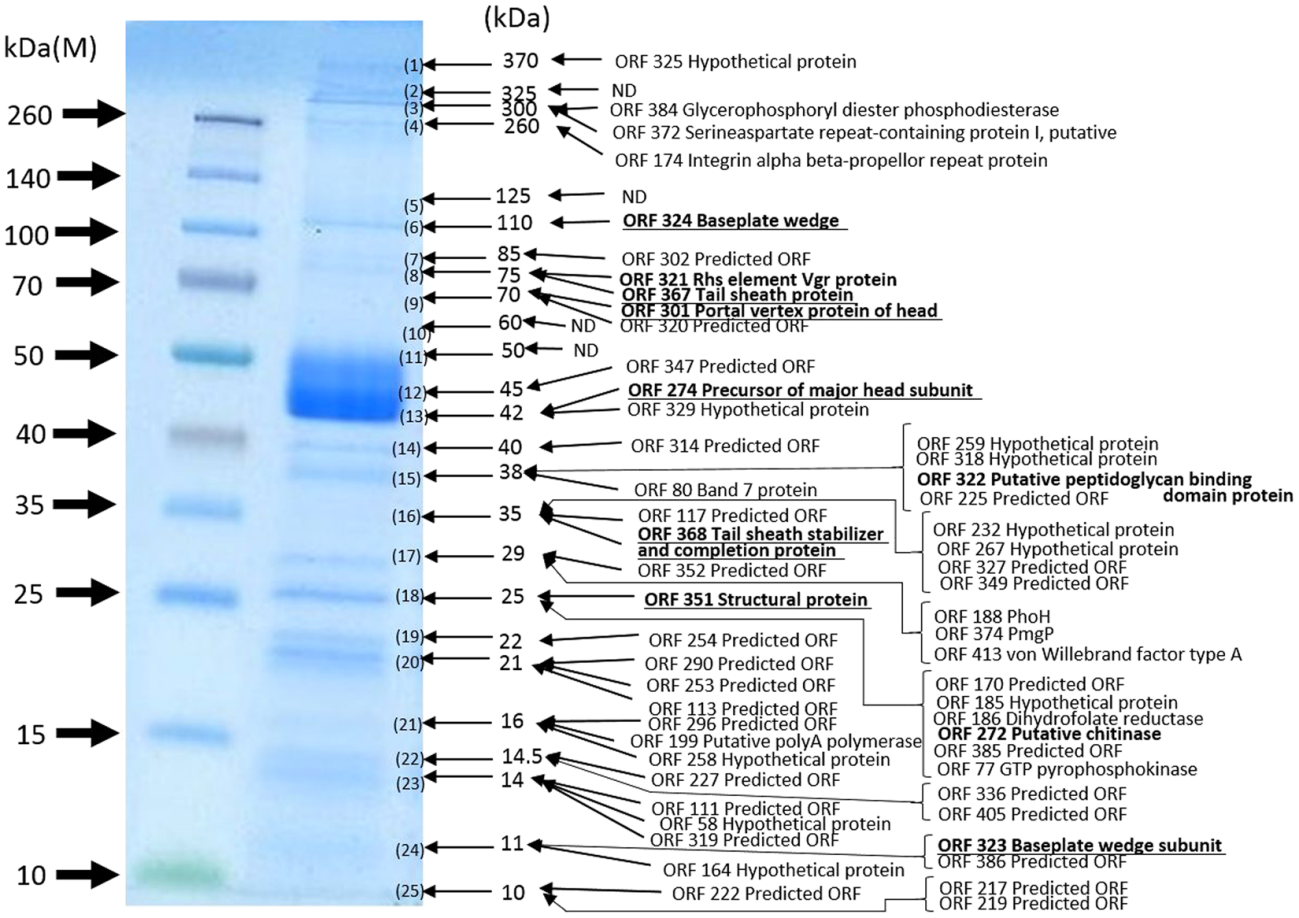
Proteomic analysis of virion proteins of XacN1. Virion proteins separated by SDS-PAGE were visualized with Coomassie Brilliant Blue. The protein bands were excised from the gel, digested with trypsin, and analyzed by liquid chromatography-tandem mass spectrometry (LTQ Orbitrap XL). Assignment of tandem mass spectrometry data to tryptic peptides encoded by phage open reading frames was completed using an established procedure^3^. ND: Not determined exactly.

The genome of XacN1 encodes potential lytic enzymes containing cell wall hydrolases (ORF112 and ORF571), M23 family peptidases (ORF118, ORF322, ORF423, and ORF577), a chitinase (ORF272), a lipase (ORF279), and a C1 family peptidase (ORF382). Among them, the chitinase (ORF272), the lipase (ORF279), and a M23 family peptidase (ORF322) were found packed in the virions according to our proteomic analysis (Fig. 3). *Xanthomonas* produces a slimy polysaccharide matrix known as xanthan gum^16^. These enzymes in the XacN1 virions may be important to break the biofilm to initiate phage infection.

XacN1 encodes stress response or toxin-antitoxin genes including GTP pyrophosphokinases (ORF77 and ORF536), a PhoH (ORF188), a phage shock protein E (ORF191), and a MazG (ORF195). While several Pho regulon genes have been identified in phages, *phoH* is the most prevalent one, appearing in 42 out of 602 completely sequenced phage genomes^41^. The existence of Pho regulon genes in phage genomes is thought to provide a selective advantage to phages, by allowing phosphate uptake during infection under phosphate limited conditions^41^. MazG is thought to act as a global transcriptional regulator through modulation of ppGpp levels, which may extend the period of cell survival under the stress of phage infection^42^.

### Structural proteins

The genome of XacN1 was predicted to encode nine structural genes (Supplementary Table S5). All the structural genes except for head completion protein (ORF348) and baseplate wedge (ORF323) had their closest homologs in Rak2-like phages. Under electron microscopy, the tail fiber structures were not observed (Fig. 1). In agreement with this observation, no tail fiber homologs were detected in the genome. These results suggest the lack of tail fibers in the virion of XacN1, while Rak2-like phages possess tail fibers. To identify structural genes of XacN1, we performed a MS/MS proteomic analysis and compared the data with predicted protein sequences. Seven of the nine annotated structural proteins were identified in the particle proteome (Fig. 3). Among predicted structural proteins in XacN1, head completion (ORF348) and baseplate hub protein (ORF317) were not detected in the MS/MS analysis. In the case of RaK2, the baseplate hub protein was also not detected by an MS/MS analysis^6^. This may be due to their low abundance in virions and to the incompatibility of these proteins with sample preparation procedures.

### XacN1 is distantly related to the Rak2-like genus

**Figure 4:**
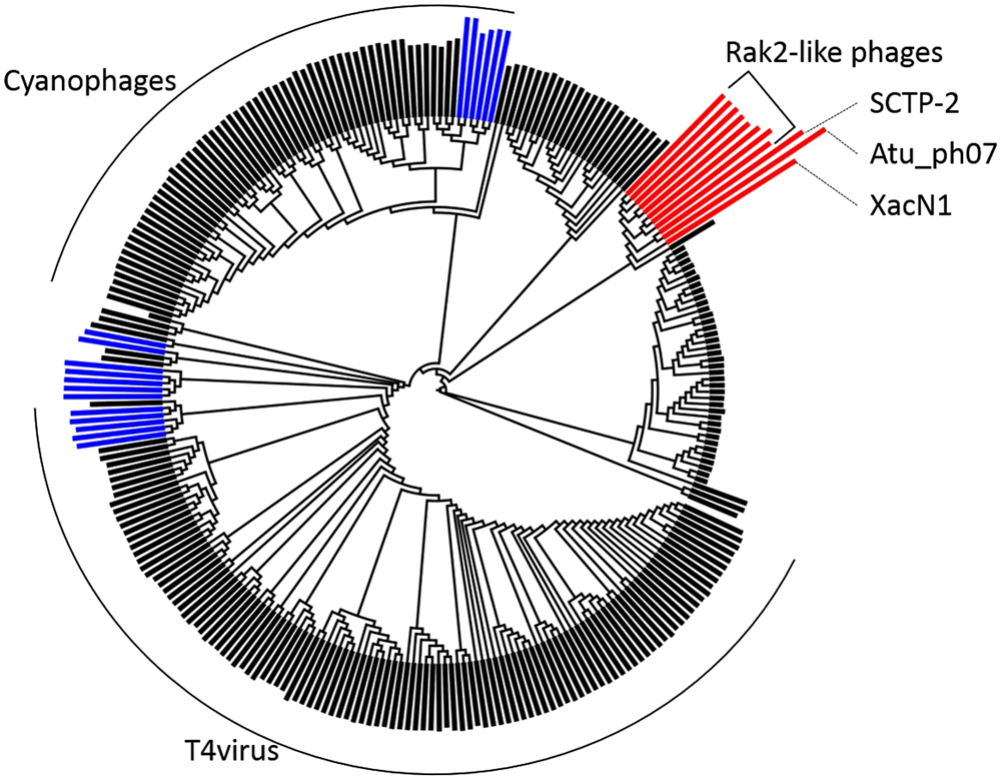
Phylogenetic relationships between XacN1 and other phages. Maximum likelihood phylogenetic trees of terminase large subunit proteins. The length of the bars outside the tree is proportional to the genome size of each phage. Red and blue bars represent genomes greater than 300 kbp and 200 kbp, respectively. Black bars represent genomes smaller than 200 kbp.

**Figure 5:**
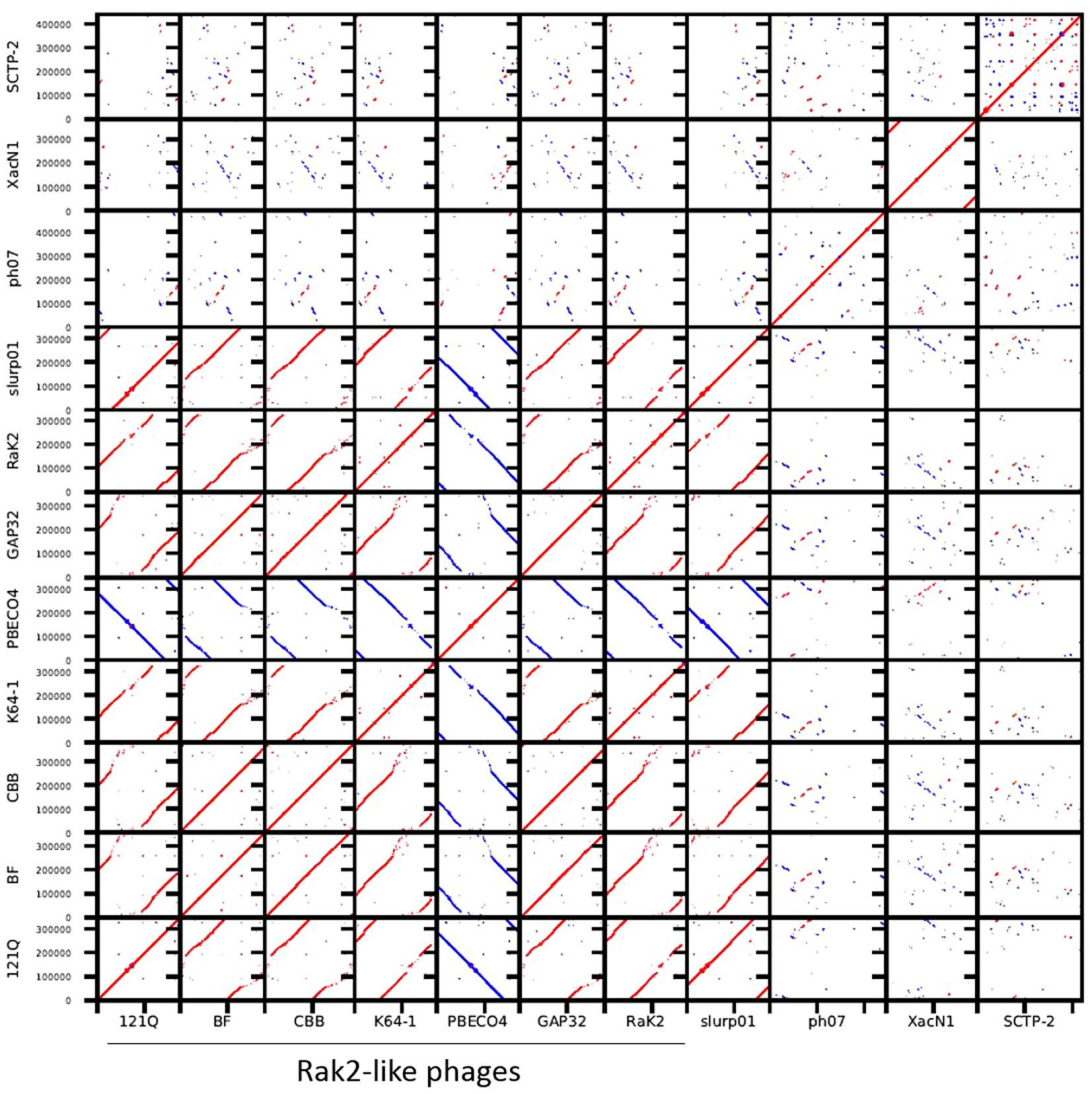
Genome comparison among eleven XacN1 related phages. Red and blue lines of the dot-plot represent sequence similarities detected by TBLASTX in the same and reverse orientations, respectively.

To investigate the evolutionary relationship between XacN1 and other phages, the phage terminase large subunit (ORF255), major capsid (ORF274), and tail sheath protein (ORF367) were used to reconstruct phylogenetic trees. Terminase (Fig. 4 and Supplementary Fig. S5A), major capsid protein (Supplementary Fig. S5B) and tail sheath protein (Supplementary Fig. S5C) trees all support a monophyletic group where XacN1 branches as the most distant lineage to ten other jumbo phages. This monophyletic group includes all seven recognized Rak2-like phages and *Escherichia* phage vB_Eco_slurp01 forming a well-supported clade, and *Agrobacterium* phage Atu_ph07 and *Salicola* phage SCTP-2 branching in-between Rak2-like and XacN1. All the phages in this clade exhibit a genome larger than 300 kbp, suggesting that a large genome size was already a feature of the ancestral phage genome of this clade. Dot-plot analysis of these phages, however, indicates substantial difference in gene order (i.e., lack of co-linearity) among the genomes of this clade (Fig. 5). Although the genome structures of members of Rak2-like phages and vB_Eco_slurp01 are well conserved, those of XacN1, Atu_ph07, and SCTP-2 show a higher level of divergence. Apart from the XacN1 genome, none of these genomic data showed long DTRs. Therefore, the large genome sizes were conserved but genome organization largely diverged in the course of the evolution of this jumbo phage clade.

To investigate genomic features specific to the phages in this monophyletic group of jumbo phages, we identified orthologous genes conserved in this group but not found in *Escherichia* phage T4 (Supplementary Table S6). The resulting list of such orthologs contained tRNA processing functions and GroES chaperonin. These jumbo phages may possess these functions, as these phages need to translate many more genes than smaller phages.

Since Rak2-like phages represent an evolutionally distinct branch of the family *Myoviridae*, it has been proposed that they form their own genus^9^. The phylogenetic relationship and the common core genes suggest that XacN1, SCTP-2, Atu_ph07, and Rak2-like phages containing vB_Eco_slurp01 form a monophyletic group. However, genomes and gene compositions were not conserved between XacN1 and Rak2-like phages. The genome-wide sequence similarity (*S*_G_) between XacN1 and other phages in the monophyletic group was 0.0282 at maximum, which is substantially lower than a previously proposed threshold for a genus-level grouping (*S*_G_ > 0.15)^37^. The genome organization and morphological characteristics of XacN1 were also vastly different from Rak2-like phages. Therefore, we propose that XacN1 represents a new genus, forming a sister group of Rak2-like phages.

### Jumbo phage genomes are enriched in translation related genes

**Figure 6:**
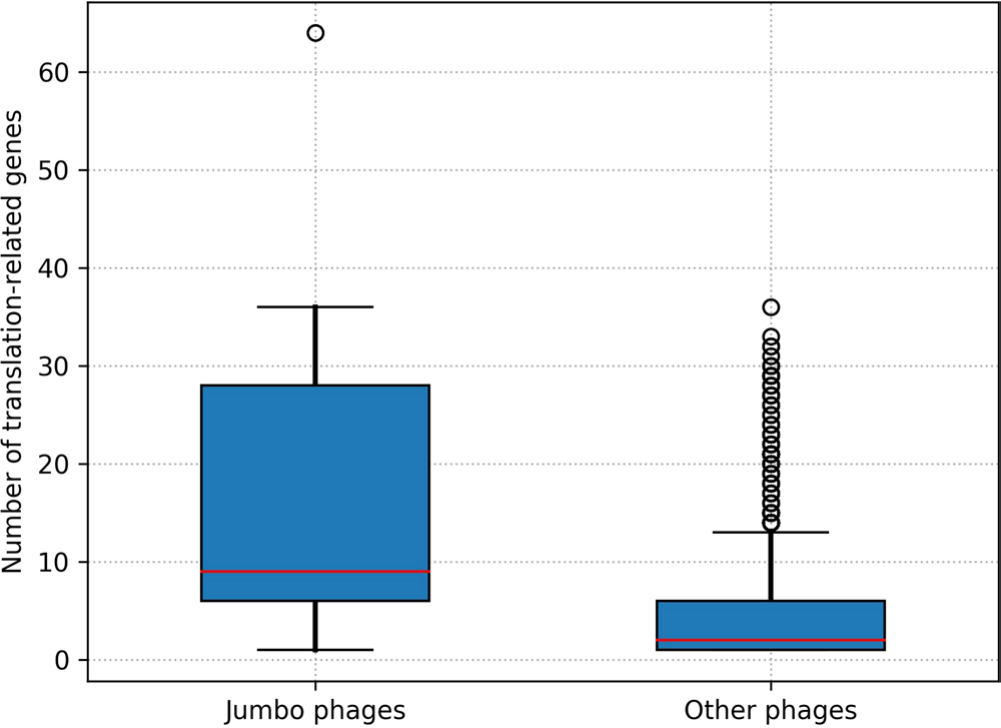
Distribution of translation-associated genes in jumbo phages and other smaller phages. Box plots represented the number of translation-associated genes including tRNA genes.

Growing evidence indicates that large DNA viruses infecting eukaryotes encode many genes for translation processes including tRNAs, aminoacyl-tRNA synthetases and other translation factors^43,44^, although no complete set of ribosomal genes has yet been identified in viruses. We examined such a tendency for phages by identifying tRNA genes and translation related enzyme genes in the currently available phage genomes. As a result, jumbo phages are found to encode a larger number of translation-related genes than smaller phages (Fig. 6; Mann–Whitney U test, *p* = 7.28 × 10^−16^). Therefore, a larger number of genes encoded in viral genomes appears to signify an increased level of independence on host translation machinery for both viruses and phages.

## Conclusion

In this work, we reported experimental and genomic investigations of *Xanthomonas citri* phage XacN1. XacN1 has a 384,670 bp linear double-stranded DNA genome with long 66 kbp direct terminal repeats. The genome encodes 56 tRNA genes, which is the most numerous among sequenced viral genomes. The comparison of codon usages between XacN1 and its host revealed a significant tendency for XacN1 to encode tRNA corresponding to codons that are more highly used by the phage than by the host genome. This result and an enrichment of tRNA processing enzyme genes suggest that protein biosynthesis of this phage is less dependent on the host compared to other smaller phages. Consistently, XacN1 showed a wider host specificity than other phages infecting *X. citri* strains. Therefore, XacN1 may be an interesting candidate towards the development of phage biocontrol of citrus canker caused by different strains of *X. citri*. Phylogenetic and comparative genomics analyses indicated that XacN1 forms a monophyletic group with ten other jumbo phages possessing genomes greater than 300 kbp.

## Methods

### Bacterial strains and culture conditions

*X. citri* strains used in this study and their origins are listed in Table S1. They were grown on nutrient agar (NA) medium (Difco, BBLBD, Cockeysville, MD, USA) at 28 °C. For preparation of bacterial suspension, *X. citri* strains were cultured for 24 h at 28 °C with shaking at 220 rpm in NB broth (Difco) according to Ahmad *et al*.^33^.

### Bacteriophage isolation and purification

Bacteriophages were isolated from a soil sample collected from orange groves in Akitsu, Higashi-Hiroshima, Japan. A 5 g soil sample was mixed with *X. citri* MAFF 301080 in 15 ml calcium carbonate medium containing 0.8% NB and 2.5% CaCO_3_ and incubated overnight at 28 °C. After centrifugation at 5,000 × g, the supernatant was filtrated through a 0.45 µm pore size filter (Steradisc, Kurabo Co., Ltd. Osaka, Japan) and subjected to plaque assay. Host bacterial cells (MAFF 301080) grown to OD_600_ = 0.3 in NB (250 µl) was added with phage sample (100 µl), mixed with 4.5 ml top agar of NB (0.3% agar), and poured onto NA plates (1.5% agar). XacN1 was detected as small clear plaques (1–2 mm). Single plaque isolation was repeated three times and purified phages were amplified with MAFF 301080 as the host. For further purification, the phage suspension was layered on a linear 20–60% sucrose gradient and centrifuged at 40,000 × g for 1 h. The purified phage was stored at 4 °C in SM buffer (50 mM Tris-HCl, at pH 7.5, 100 mM NaCl, 10 M MgSO_4_, and 0.01% gelatin). For electron microscopy, the phage particles were stained with 1% ammonium molybdate (pH 7.5) and observed in a JEOL JEM-1400 electron microscope (JEOL Ltd., Tokyo, Japan). Lambda phage particles were used as an internal standard marker for size determination. An icosahedral head (diameter: 129 ± 10 nm, n = 10) and a contractile tail (length: 128 ± 5.0 nm, n = 10; width: 15.3 ± 2.5 nm, n = 10, respectively) were observed.

### Host range determination and single-step growth experiments

For host range determination, initially lysis zone formation spot tests^45^ and then standard plaque-forming assays^33^ were performed with strains listed in Table S1 as the hosts. Single-step growth experiments were performed as previously described^33^, with some modifications. Strains MAFF 301080 was used as the host. Cells (0.1 U of OD_600_) were harvested by centrifugation and resuspended in fresh NB (ca. 1 × 10^8^ CFU/ml) to a final culture volume of 10 ml. Phage was added at an MOI of 1.0 and allowed to adsorb for 10 min at 28 °C. After centrifugation and resuspending in the initial volume of NB with decimal dilution to a final volume of 10 ml, the cells were incubated at 28 °C. Samples were taken at 30-min intervals and immediately diluted with or without chloroform treatment, and the titers were determined by the double-layered agar plate method.

### Identification of virion proteins by liquid chromatography-tandem mass spectrometry

Phage structural proteins separated by SDS-polyacrylamide gel electrophoresis (SDS-PAGE) (4–15% polyacrylamide) were stained with Coomassie Brilliant Blue, excised from the in-gel and digested with trypsin. The tryptic peptides were trapped with a short ODS column (PepMap 100; 5 × 0.3 mm ID, Thermo Fisher Scientific Inc. as described before^4^, Waltham, MA, USA, 30 µL/min) and separated with a long ODS column (Capillary Column; 120 × 0.075 mm ID, Nikkyo Technos, Tokyo, Japan, 0.2 µL/min) using nano-liquid chromatography (Ultimate 3000 RSLC, Thermo Fisher Scientific Inc.). The eluate was continuously introduced into a nanoESI source and analyzed by mass spectrometry (MS) and MS/MS (LTQ Orbitrap XL, Thermo Fisher Scientific Inc.). The MS and MS/MS spectra were generated in the positive ion mode using Orbitrap (*m/z* 300–1500) and Iontrap (data-dependent scan of top five peaks using CID), respectively, with the capillary source voltage at 1.5 kV and the transfer capillary temperature at 200 °C. The MS/MS data was assigned to tryptic peptides encoded by ORFs as described before^4^ with the Xcalibur program ver. 2.0 (Thermo Fisher Scientific Inc.). All MS/MS data were searched thorough the GenBank non-redundant protein database and an in-house database of all possible XacN1 gene product using Mascot (Matrix Science KK, Tokyo, Japan). Doubly, triply, and quadruply charged peptide ions were subjected to the database search with precursor and fragment ion mass tolerance of ±10 ppm and ±0.8 Da, respectively, with static modification (carbamidomethylation of cysteine) and dynamic modification (oxidation of methionine and deamidation of asparagine and glutamine). The significance threshold on Proteome Discoverer for Mascot search was set at P < 0.05 and one and two missed trypsin cleavage was allowed. Proteomics raw data and search files for protein identification of XacN1 have been deposited to the ProteomeXchange Consortium (announced ID: PXD008065) via the jPOST partner repository (announced ID: JPST000339).

### Isolation and sequencing of genomic DNA from XacN1

DNA extraction, purification, digestion with restriction enzymes, and sequencing were performed according to Sambrook and Russel^46^. The whole genome size of XacN1 was determined by pulsed-field gel electrophoresis (PFGE) according to Higashiyama and Yamada^47^. Briefly, after purification, XacN1 particles were embedded in 1% low-melting-point agarose (InCert agarose; FMC Corp., Philadelphia, PA, USA). Phage-containing plugs were treated by proteinase K (1 mg/mL Merck Ltd., Tokyo, Japan) and 1% Sarkosyl, and subjected to (PFGE) by a using a CHEF Mapper electrophoresis apparatus (Bio-Rad, Hercules, CA, USA). For sequencing, genomic DNA was extracted from the purified phage particles by phenol extraction. Shotgun sequencing of the DNA was performed at Hokkaido System Science Co., Ltd. (Sapporo, Japan) by using a PacBio RSII System (http://www.pacb.com/products -and-services/pacbio-systems/rsii/). XacN1 genomic sequences were assembled into a linear contig of 318,795 bp by using a SMART Analysis software (ver. 2.3.0.140936.p4.150482) (http://www.pacb.com/products-and-services/analytical-software/smart-analysis/). This size was approximately 70 kbp smaller than that determined by PFGE (Supplementary Fig. S1). Detailed examination in sequence coverage through the entire region showed that a 70 kb region on the right end of the contig had a coverage of approximately 2000X while the remaining region was approximately 1000X (Supplementary Fig. S5A and B). This suggested that a 70 kbp duplicated region was collapsed during the initial assembly. The length of the genome assembly and the size of the inferred duplicated region coincided with the genome size as determined by PFGE (~390 kbp). Among several patterns of duplication of this region on the XacN1 genome, we determined the most probable one by restriction enzyme digestion analysis of the genomic DNA with *Bss*HII, *Nae*I, and *Spe*I (Supplementary Fig. S5C). The exact right end and the left end of linear XacN1 genomic DNA were determined by direct sequencing using PCR with primers 5′GCC ACC ACA GCA GAT AGG ACG ATA CCC GTG (positions 384,005–384,034, forward) and 5′ TTA GGG GTT GAC ATT TGT CAG CCC CTT TTG (positions 781–810, reverse), respectively (Supplementary Fig. S5D and E). The sequence at the border between repeats and the unique region was also determined by PCR in the same way with corresponding genomic fragments as the templates. The resulting final size of XacN1 genomic DNA was 384,670 bp with repetition of a 65,875 bp-sequence at both ends (DDBJ accession number: AP018399).

ORFs were predicted with GeneMarkS (version 4.32)^48^. Amino acid sequences of each ORF were searched for homologs against RefSeq (Release 83)^49^ and CDD (version 3.16)^50^ using BLASTP^51^ and RPS-BLAST^52^ of BLAST+ (version 2.6.0)^53^. An E-value lower than 1e-5 was used as the cutoff for notable similarity. Function annotation was then performed by manual investigation of the homology search results. tRNAscan-SE (version 1.3.1)^54^ was used to search for tRNA genes. Translation-associated genes of phages were detected by BLAST best-hit ortholog groups using KO (KEGG Orthology)^55^ and Clusters of Orthologous Groups (COG)^56^ database. With these computationally detected genes, additional translation-associated genes in phage genomes were manually identified based on gene function annotations.

### Codon usage analysis

To investigate an adaptive role of phage-encoded tRNAs, codon usages were calculated for XacN1 and its host (strain 306). Codon usages were determined based on phage and host CDSs (coding sequences). For each of 64 codons, a relative codon frequency, *f*_*i*_, was calculated using the following formula.

$${{f}}_{{i}}=\frac{{Number}\,{of}\,{codon}\,{i}}{{Number}\,{of}\,{all}\,{codons}}$$

The ratio of the relative codon frequencies, *r*_*i*_, between the phage and host were then computed using the following formula.

$${{r}}_{{i}}=\frac{{{f}}_{{i}}^{{PHAGE}}}{{{f}}_{{i}}^{{HOST}}}$$

Codons were then categorized into three groups according to the following criteria. If *r*_*i*_ is 1.1 or greater, the phage exhibits a higher relative codon frequency for codon *i* than the host. If *r*_*i*_ is 0.9 or greater and smaller than 1.1, the phage and the host exhibit similar relative codon frequencies. If *r*_*i*_ is smaller than 0.9, the host exhibits a higher relative codon frequency. We considered only those codons that were complementary to the anticodons of the tRNAs encoded by the phage or host (Supplementary Table S4). Finally, the relationship between these three groups of codons and the presence of the corresponding tRNA genes in the phage genome was tested using Fisher’s exact test.

### Phylogenetic and core gene analysis

Homologs of terminase large subunit, major capsid, and tail sheath proteins were recruited using PSI-BLAST^57^ against the RefSeq sequence database and some additional phages. An E-value lower than 1e-5 was used as the cutoff for notable similarity. Sequences were aligned using MAFFT (v7.220)^58^ with default parameters. Tree reconstruction was performed using RAxML (v8.2.4)^59^ with the selected LG + F substitution model and PROTGAMMA parameter with 100 bootstrap replicates. Dot plot was generated by an in-house script (available at http://mbi3.kuicr.kyoto-u.ac.jp/supp/yos/XacN1/). Clusters of *Myoviridae* orthologous genes were identified by grouping genes in all available *Myoviridae* genomes using OrthoFinder (v1.1.10)^60^. Core genes of the XacN1-Rak2-like clade were defined as orthologs present in XacN1 and at least one other member of this clade. From this core gene set, we subtracted orthologs present in T4 in order to define the set of genes specific to the XacN1-Rak2-like clade.

## Electronic supplementary material

Supplementary information
