# Supplementary material for: Xanthomonas citri jumbo phage XacN1 exhibits a wide host range and high complement of tRNA genes
Source: Sci Rep. 2018 Mar 14;8:4486. doi: 10.1038/s41598-018-22239-3 (PMC5852040; doi:10.1038/s41598-018-22239-3)
Supplement: Supplementary file 1 — Supplementary information [file 41598_2018_22239_MOESM1_ESM.pdf]

Supplementary Information

Xanthomonas citri jumbo phage XacN1 exhibits a wide host range and high complement of tRNA genes.

Genki Yoshikawa[1], Ahmed Askora[2,3], Romain Blanc-Mathieu[1], Takeru Kawasaki[2], Yanze Li[1], Miyako Nakano[2], Hiroyuki Ogata[1,*], Takashi Yamada[2,4,*]

[1] Institute for Chemical Research, Kyoto University, Gokasho, Uji 611-0011, Japan
[2] Department of Molecular Biotechnology, Graduate School of Advanced Sciences of Matter, Hiroshima University, Higashi-Hiroshima 739-8530, Japan.
[3] Department of Microbiology, Faculty of Science, Zagazig University, 44519 Zagazig, Egypt
[4] Hiroshima Study Center, The Open University of Japan, Hiroshima 730-0053, Japan

[*] Co-corresponding authors: ogata@kuicr.kyoto-u.ac.jp, tayamad@hiroshima-u.ac.jp

Supplementary Table S1. Host range of XacN1

Xanthomonas citri strain	Host plant	Bacterial source	XacN1	ϕCp1[a]	ϕCp[a]
MAFF 301077[b]	*Citrus limon*	NIAS[c]	+[d]	+	-
MAFF 301080	*C. sinensis*	NIAS	+	+	-
MAFF 302102	*Citrus sp.*	NIAS	+	-	+
MAFF 673001	*C. natsudaidai*	NIAS	+	-	+
MAFF 673010	*Citrus sp.*	NIAS	+	-	+
MAFF 673011	*C. limon*	NIAS	-	+	-
MAFF 673013	*Citrus sp.*	NIAS	+	+	-
MAFF 673018	*Citrus sp.*	NIAS	+	-	+
MAFF 673021	*C. limon*	NIAS	+	-	+
KC33	*C. iyo*	Shiotani *et al.*, 2007[e]	+	+	+

[a] Bacteriophages Cp1 and Cp2 are standard phages used for typing of *Xanthomonas citri* strains (Ahmad *et al.*, 2013).
[b] MAFF, Ministry of Agriculture, Forestry and Fishery, Japan.
[c] NIAS, National Institute for Agrobiological Sciences, Japan.
[d] Sensitivity: +, sensitive (EOP>10^{-2} pfu/plate); -, resistant (EOP<10^{-6} pfu/plate). An EOP of 1 was equivalent to 333 pfu/plate.
[e] Shiotani, H., Fujikawa, T., Ishihara, H., Tsuyumu, S. & Ozaki, K. A pthA homolog from *Xanthomonas axonopodis* pv. citri responsible for host-specific suppression of virulence. *J. Bacteriol.* **189,** 3271ï 3279 (2007).

Supplementary Table S2. Annotation of XacN1 ORFs.

ORF	strand	start	end	length (aa)	Annotation
ORF1	-	92	325	77	Predicted ORF
ORF2	-	381	743	120	Predicted ORF
ORF3	-	829	1218	129	Hypothetical protein
ORF4	-	1227	1409	60	Predicted ORF
ORF5	-	1469	1732	87	Hypothetical protein
ORF6	-	1855	2046	63	Predicted ORF
ORF7	-	2046	2171	41	Predicted ORF
ORF8	+	2240	2461	73	Predicted ORF
ORF9	-	2471	2860	129	Hypothetical protein
ORF10	-	2899	3186	95	Predicted ORF
ORF11	-	3338	3574	78	Predicted ORF
ORF12	-	3576	4250	224	Predicted ORF
ORF13	-	4303	4704	133	Predicted ORF
ORF14	-	4757	5155	132	Hypothetical protein
ORF15	-	5348	5647	99	Predicted ORF
ORF16	-	5695	6039	114	Predicted ORF
ORF17	-	6041	6244	67	Predicted ORF
ORF18	-	6296	6514	72	Predicted ORF
ORF19	-	6511	6678	55	Predicted ORF
ORF20	-	6716	7009	97	Hypothetical protein
ORF21	-	7006	7203	65	Predicted ORF
ORF22	-	7203	7529	108	Predicted ORF
ORF23	-	7560	7673	37	Predicted ORF
ORF24	-	7733	7963	76	Predicted ORF
ORF25	-	8158	8388	76	Predicted ORF
ORF26	-	8391	8549	52	Predicted ORF
ORF27	-	8621	8839	72	Predicted ORF
ORF28	-	9407	9640	77	Predicted ORF
ORF29	-	9689	10009	106	Hypothetical protein
ORF30	-	10009	10188	59	Hypothetical protein
ORF31	-	10188	10424	78	Hypothetical protein
ORF32	-	10421	10561	46	Predicted ORF
ORF33	-	10616	11224	202	Hypothetical protein
ORF34	-	11436	11711	91	Predicted ORF
ORF35	-	11762	11869	35	Predicted ORF
ORF36	-	11980	12171	63	Predicted ORF
ORF37	-	12239	12469	76	Predicted ORF
ORF38	-	12471	12755	94	Hypothetical protein
ORF39	-	12917	13420	167	Hypothetical protein
ORF40	-	13575	13790	71	Predicted ORF
ORF41	-	13911	14654	247	Predicted ORF
ORF42	-	14731	14931	66	Predicted ORF
ORF43	-	15172	15504	110	Hypothetical protein
ORF44	-	15566	15931	121	Predicted ORF
ORF45	-	15996	16511	171	SprT-like protein
ORF46	-	16572	16823	83	Predicted ORF
ORF47	-	16988	17254	88	Predicted ORF
ORF48	-	17450	17695	81	Predicted ORF
ORF49	-	17708	17872	54	Predicted ORF
ORF50	-	17932	18339	135	Predicted ORF
ORF51	-	18336	18566	76	Hypothetical protein
ORF52	-	18737	18994	85	Predicted ORF
ORF53	-	19111	19290	59	Predicted ORF
ORF54	-	19298	19639	113	Hypothetical protein
ORF55	-	19658	19855	65	Predicted ORF
ORF56	-	19864	20172	102	Predicted ORF
ORF57	-	20239	20451	70	Predicted ORF
ORF58	+	20560	20967	135	Hypothetical protein
ORF59	-	20964	21266	100	Hypothetical protein
ORF60	-	21326	21550	74	Predicted ORF
ORF61	-	21547	21711	54	Predicted ORF
ORF62	-	21744	22124	126	HNH endonuclease
ORF63	-	22135	22383	82	Predicted ORF
ORF64	+	22559	23008	149	N-acetyltransferase
ORF65	+	23257	23778	173	Predicted ORF
ORF66	+	23827	24075	82	Hypothetical protein
ORF67	+	24075	24311	78	Predicted ORF
ORF68	+	24351	24704	117	Predicted ORF
ORF69	+	24822	25187	121	Predicted ORF

ORF70	+	25315	26067	250	Hypothetical protein
ORF71	+	26116	26622	168	Hypothetical protein
ORF72	+	26632	26811	59	Predicted ORF
ORF73	+	26869	27306	145	Predicted ORF
ORF74	+	27306	27887	193	Hypothetical protein
ORF75	+	27889	28188	99	Predicted ORF
ORF76	+	28223	28627	134	Predicted ORF
ORF77	+	28637	29275	212	GTP pyrophosphokinase
ORF78	+	29281	29934	217	Predicted ORF
ORF79	+	30045	30269	74	Predicted ORF
ORF80	+	31115	32026	303	Band 7 protein
ORF81	+	32134	32553	139	Predicted ORF
ORF82	+	32638	34299	553	Hypothetical protein
ORF83	+	34317	34673	118	Predicted ORF
ORF84	+	34670	34912	80	Predicted ORF
ORF85	+	34945	35139	64	Predicted ORF
ORF86	+	35180	35491	103	Predicted ORF
ORF87	+	35501	36040	179	Hypothetical protein
ORF88	+	36246	36560	104	Hypothetical protein
ORF89	+	39846	40211	121	Predicted ORF
ORF90	+	40262	40540	92	Predicted ORF
ORF91	+	40885	41613	242	Predicted ORF
ORF92	+	41610	41813	67	Predicted ORF
ORF93	+	41971	42198	75	Hypothetical protein
ORF94	+	42185	42319	44	Predicted ORF
ORF95	+	42695	42952	85	Predicted ORF
ORF96	+	43024	43299	91	Predicted ORF
ORF97	+	43420	43626	68	Predicted ORF
ORF98	+	43856	44104	82	Predicted ORF
ORF99	+	44212	44601	129	Predicted ORF
ORF100	+	44611	44982	123	Predicted ORF
ORF101	+	45467	45736	89	Predicted ORF
ORF102	+	45757	45966	69	Predicted ORF
ORF103	+	46705	47115	136	Predicted ORF
ORF104	+	47115	47396	93	Predicted ORF
ORF105	+	47622	47780	52	Predicted ORF
ORF106	+	47944	48630	228	Predicted ORF
ORF107	+	49048	49260	70	Predicted ORF
ORF108	+	49586	49828	80	Predicted ORF
ORF109	+	50040	50420	126	Predicted ORF
ORF110	+	50601	51215	204	Predicted ORF
ORF111	+	51240	51602	120	Predicted ORF
ORF112	+	51691	52290	199	cell wall hydrolase
ORF113	+	52295	52909	204	Predicted ORF
ORF114	+	52974	53399	141	Predicted ORF
ORF115	+	53496	53693	65	Predicted ORF
ORF116	+	53725	53925	66	Predicted ORF
ORF117	-	53927	54754	275	Predicted ORF
ORF118	-	54866	55600	244	M23 family peptidase
ORF119	-	55719	56567	282	Hypothetical protein
ORF120	-	56564	56779	71	Predicted ORF
ORF121	-	56776	58083	435	RNA ligase
ORF122	-	58197	59198	333	protein rIIB
ORF123	-	59264	61471	735	protein rIIA
ORF124	-	61743	61904	53	Predicted ORF
ORF125	-	61966	62316	116	Predicted ORF
ORF126	-	62303	63052	249	$tRNA^{His}$ guanylyltransferase
ORF127	-	63052	63588	178	Predicted ORF
ORF128	-	63588	63851	87	Predicted ORF
ORF129	-	63907	64128	73	Predicted ORF
ORF130	-	64163	64510	115	Predicted ORF
ORF131	-	64507	64683	58	Predicted ORF
ORF132	-	64716	65024	102	Predicted ORF
ORF133	-	65047	65391	114	Predicted ORF
ORF134	+	66126	66446	106	Predicted ORF
ORF135	+	66481	66810	109	Predicted ORF
ORF136	+	66810	67181	123	Predicted ORF
ORF137	+	67276	67617	113	Predicted ORF
ORF138	+	67617	67925	102	Predicted ORF
ORF139	+	67964	68365	133	Predicted ORF
ORF140	+	68378	68764	128	Predicted ORF
ORF141	+	68764	69177	137	Predicted ORF

ORF142	+	69209	69598	129	Predicted ORF
ORF143	+	69623	70102	159	Predicted ORF
ORF144	+	70099	70485	128	Predicted ORF
ORF145	+	70482	70868	128	Predicted ORF
ORF146	+	70903	71340	145	Predicted ORF
ORF147	+	71475	71801	108	Predicted ORF
ORF148	+	71891	72493	200	Predicted ORF
ORF149	+	72480	73013	177	Predicted ORF
ORF150	+	73051	73497	148	Predicted ORF
ORF151	+	73542	74138	198	Predicted ORF
ORF152	+	74135	74554	139	Predicted ORF
ORF153	+	74591	75346	251	FkbM family methyltransferase
ORF154	+	75349	75843	164	Predicted ORF
ORF155	+	75852	76034	60	Predicted ORF
ORF156	+	76034	76285	83	Hypothetical protein
ORF157	+	76282	76872	196	Hypothetical protein
ORF158	+	76869	77189	106	Predicted ORF
ORF159	+	77186	77509	107	Predicted ORF
ORF160	-	77524	77697	57	Predicted ORF
ORF161	-	77697	78347	216	Predicted ORF
ORF162	+	78456	78752	98	Predicted ORF
ORF163	+	78745	79011	88	Predicted ORF
ORF164	+	79061	79381	106	DNA-binding protein
ORF165	+	79501	79740	79	glutaredoxin
ORF166	+	79745	79972	75	glutaredoxin
ORF167	+	80083	80529	148	Appr-1-p processing protein
ORF168	+	80587	80769	60	Predicted ORF
ORF169	+	80780	80977	65	Predicted ORF
ORF170	-	81006	81653	215	Predicted ORF
ORF171	-	81653	82057	134	Predicted ORF
ORF172	+	82159	82662	167	5' nucleotidase
ORF173	+	82662	83066	134	GNAT family N-acetyltransferase
ORF174	-	83103	84863	586	Hypothetical protein
ORF175	-	84910	85101	63	Predicted ORF
ORF176	-	85221	86102	293	DNA primase
ORF177	-	86288	87001	237	Clp protease
ORF178	-	87166	89079	637	NAD-dependent DNA ligase
ORF179	+	89172	89435	87	Predicted ORF
ORF180	+	89435	93214	1259	Hypothetical protein
ORF181	+	93282	95153	623	DNA topoisomerase II large subunit
ORF182	+	95150	96475	441	DNA topoisomerase II medium subunit
ORF183	-	96671	96823	50	Predicted ORF
ORF184	-	96804	97001	65	Predicted ORF
ORF185	-	97001	97573	190	Hypothetical protein
ORF186	-	97583	98137	184	dihydrofolate reductase
ORF187	-	98139	98420	93	Predicted ORF
ORF188	+	98733	99512	259	PhoH
ORF189	+	99601	99912	103	Predicted ORF
ORF190	+	99915	100556	213	Predicted ORF
ORF191	-	100587	100871	94	phage shock protein E
ORF192	-	100944	101378	144	Predicted ORF
ORF193	-	101381	101962	193	Hypothetical protein
ORF194	-	101959	102222	87	Predicted ORF
ORF195	-	102232	102834	200	MazG nucleotide pyrophosphohydrolase
ORF196	-	102836	103021	61	Predicted ORF
ORF197	-	103018	104232	404	metallophosphoesterase
ORF198	-	104235	105524	429	methyltransferase
ORF199	-	105598	106092	164	predicted kinase
ORF200	-	106154	106708	184	alkylated DNA repair dioxygenase
ORF201	-	106705	106980	91	Predicted ORF
ORF202	-	106965	107495	176	methyltransferase
ORF203	-	107528	108118	196	Hypothetical protein
ORF204	-	108168	108902	244	deoxycytidylate deaminase
ORF205	-	108902	109249	115	Predicted ORF
ORF206	-	109317	110729	470	nicotinate phosphoribosyltransferase
ORF207	-	110872	111369	165	N-acetyltransferase
ORF208	-	111369	111980	203	Hypothetical protein
ORF209	-	111983	112189	68	Predicted ORF
ORF210	-	112167	112304	45	Predicted ORF
ORF211	-	112288	112845	185	Hypothetical protein
ORF212	-	112845	113012	55	Predicted ORF
ORF213	-	112996	113583	195	DUF1768 domain containing protein

ORF214	-	113580	113987	135	Hypothetical protein
ORF215	-	114045	114311	88	Predicted ORF
ORF216	-	114321	115424	367	bifunctional nicotinamide mononucleotide adenylyltransferase/ADP-ribose pyrophosphatase
ORF217	+	115522	115839	105	Predicted ORF
ORF218	-	116064	117320	418	Predicted ORF
ORF219	-	117330	117659	109	Predicted ORF
ORF220	-	117656	118036	126	Predicted ORF
ORF221	-	118033	118455	140	Predicted ORF
ORF222	-	118502	118786	94	Predicted ORF
ORF223	-	118779	119372	197	DNA polymerase III epsilon subunit
ORF224	-	119369	119977	202	deoxynucleoside monophosphate kinase
ORF225	-	120041	120733	230	Predicted ORF
ORF226	+	120842	121279	145	Predicted ORF
ORF227	-	121272	121676	134	Predicted ORF
ORF228	-	121673	122239	188	Hypothetical protein
ORF229	-	122271	122951	226	Predicted ORF
ORF230	-	122951	123118	55	Predicted ORF
ORF231	-	123176	124033	285	Hypothetical protein
ORF232	-	124048	125634	528	Hypothetical protein
ORF233	-	125682	126974	430	Hypothetical protein
ORF234	-	126993	128288	431	Hypothetical protein
ORF235	-	128304	129608	434	Hypothetical protein
ORF236	-	129620	131860	746	Hypothetical protein
ORF237	-	131899	133104	401	Hypothetical protein
ORF238	-	133143	133832	229	Hypothetical protein
ORF239	-	133829	134281	150	Predicted ORF
ORF240	-	134278	134607	109	Predicted ORF
ORF241	-	134607	135539	310	sliding clamp loader subunit
ORF242	-	135593	136438	281	Hypothetical protein
ORF243	-	136438	136749	103	Predicted ORF
ORF244	-	136752	137102	116	Predicted ORF
ORF245	-	137136	137684	182	DNA polymerase III alpha subunit
ORF246	-	137684	138133	149	Hypothetical protein
ORF247	-	138149	138637	162	Predicted ORF
ORF248	-	138665	140206	513	helicase
ORF249	-	140187	140651	154	Predicted ORF
ORF250	-	140648	141241	197	Predicted ORF
ORF251	-	141297	142430	377	RecA-like protein
ORF252	-	142498	143511	337	single-stranded DNA binding protein
ORF253	-	143655	144191	178	Predicted ORF
ORF254	-	144191	144736	181	Predicted ORF
ORF255	-	144762	146408	548	terminase large subunit
ORF256	-	146458	147186	242	Predicted ORF
ORF257	-	147240	148157	305	Hypothetical protein
ORF258	-	148473	149033	186	Hypothetical protein
ORF259	-	149054	150244	396	Hypothetical protein
ORF260	-	150606	151256	216	2'-5' RNA ligase
ORF261	-	151297	152082	261	Hypothetical protein
ORF262	-	152082	152474	130	Predicted ORF
ORF263	-	152526	154220	564	putative T5 A1-like protein
ORF264	-	154236	154730	164	Predicted ORF
ORF265	-	154754	155011	85	Predicted ORF
ORF266	-	155021	156580	519	DNA primase/helicase
ORF267	-	156666	158321	551	Hypothetical protein
ORF268	-	158336	161269	977	Hypothetical protein
ORF269	-	161306	161956	216	Hypothetical protein
ORF270	-	162027	162356	109	Predicted ORF
ORF271	-	162358	162627	89	Predicted ORF
ORF272	-	162627	163262	211	chitinase
ORF273	-	163384	163794	136	Predicted ORF
ORF274	-	163897	165288	463	major capsid protein
ORF275	-	165358	166314	318	Hypothetical protein
ORF276	+	166518	166763	81	Predicted ORF
ORF277	+	166788	167048	86	Predicted ORF
ORF278	-	167045	167221	58	Predicted ORF
ORF279	-	167196	167909	237	lipase
ORF280	-	167944	168246	100	Predicted ORF
ORF281	-	168239	168412	57	Predicted ORF
ORF282	-	168487	168786	99	co-chaperone GroES
ORF283	-	168783	168989	68	Predicted ORF
ORF284	-	169041	169484	147	Predicted ORF
ORF285	-	169459	169737	92	Predicted ORF

ORF286	-	169734	170186	150	Hypothetical protein
ORF287	-	170246	170671	141	putative NUDIX hydrolase
ORF288	-	170668	171207	179	Hypothetical protein
ORF289	-	171204	171644	146	YbiA-like protein
ORF290	+	171715	172302	195	Hypothetical protein
ORF291	-	172333	172518	61	FmdB family protein
ORF292	-	172526	172771	81	Predicted ORF
ORF293	-	172828	173391	187	phosphoesterase
ORF294	-	173405	174010	201	Hypothetical protein
ORF295	-	174042	174458	138	peptidyl-tRNA hydrolase
ORF296	+	174593	175045	150	Predicted ORF
ORF297	-	175133	175759	208	prohead core scaffolding protein and protease
ORF298	-	175764	176159	131	Predicted ORF
ORF299	-	176169	176411	80	Predicted ORF
ORF300	-	176389	176994	201	Predicted ORF
ORF301	-	177017	178756	579	portal vertex protein
ORF302	-	178807	181563	918	Predicted ORF
ORF303	+	181678	182271	197	late transcription sigma factor
ORF304	-	182255	182470	71	Predicted ORF
ORF305	-	182467	185766	1099	recombination endonuclease subunit
ORF306	-	185777	186253	158	Predicted ORF
ORF307	-	186250	186795	181	Predicted ORF
ORF308	-	186792	187145	117	Hypothetical protein
ORF309	-	187129	187248	39	Predicted ORF
ORF310	-	187250	187657	135	Predicted ORF
ORF311	-	187654	187995	113	Predicted ORF
ORF312	-	187998	189677	559	ATP-dependent helicase
ORF313	+	189691	189852	53	Predicted ORF
ORF314	-	190362	191417	351	Predicted ORF
ORF315	-	191441	192175	244	Predicted ORF
ORF316	-	192175	192357	60	Predicted ORF
ORF317	-	192381	193220	279	baseplate hub subunit
ORF318	+	193321	194109	262	Hypothetical protein
ORF319	+	194115	194474	119	Predicted ORF
ORF320	+	194471	196318	615	Predicted ORF
ORF321	+	196318	199098	926	Hypothetical protein
ORF322	+	199113	199952	279	M23 family peptidase
ORF323	+	199971	200348	125	baseplate wedge
ORF324	+	200389	203304	971	baseplate wedge
ORF325	+	203370	213311	3313	Hypothetical protein
ORF326	+	213308	213829	173	Hypothetical protein
ORF327	+	213850	214563	237	Predicted ORF
ORF328	+	214560	215228	222	Glycosyltransferase family 9
ORF329	+	215248	216456	402	Hypothetical protein
ORF330	-	216551	218191	546	Hypothetical protein
ORF331	-	218195	218935	246	Predicted ORF
ORF332	-	218928	219788	286	Predicted ORF
ORF333	-	219791	222088	765	carbamoyl transferase
ORF334	-	222135	223508	457	glycosyltransferase family protein
ORF335	+	223631	224068	145	Predicted ORF
ORF336	-	224105	224647	180	Predicted ORF
ORF337	+	224722	225153	143	NUDIX domain containing protein
ORF338	-	225125	226054	309	Hypothetical protein
ORF339	+	226090	226458	122	Hypothetical protein
ORF340	-	226466	227416	316	RnaseH
ORF341	-	227416	228027	203	Predicted ORF
ORF342	-	228037	228768	243	Predicted ORF
ORF343	-	228783	231431	882	DNA polymerase
ORF344	-	231440	231766	108	Predicted ORF
ORF345	-	231829	232437	202	Predicted ORF
ORF346	-	232430	232897	155	Predicted ORF
ORF347	-	232894	234300	468	Predicted ORF
ORF348	-	234338	234817	159	head completion protein
ORF349	-	234817	235596	259	Predicted ORF
ORF350	-	235607	237571	654	Hypothetical protein
ORF351	-	237647	238231	194	structural protein
ORF352	+	238329	239063	244	Predicted ORF
ORF353	-	239082	239774	230	Predicted ORF
ORF354	+	239851	240474	207	Hypothetical protein
ORF355	+	240474	240692	72	Predicted ORF
ORF356	+	240685	241425	246	NAD+ synthetase
ORF357	+	241493	241729	78	Hypothetical protein

ORF358	+	241716	241943	75	Predicted ORF
ORF359	+	241943	242128	61	Predicted ORF
ORF360	+	242125	242568	147	glutamyl-tRNA amidotransferase
ORF361	+	242565	242912	115	Predicted ORF
ORF362	+	242909	243097	62	Predicted ORF
ORF363	+	243098	243622	174	Hypothetical protein
ORF364	+	243756	244595	279	Hypothetical protein
ORF365	+	244612	245283	223	Predicted ORF
ORF366	-	245313	246131	272	Hypothetical protein
ORF367	-	246272	248416	714	tail sheath protein
ORF368	-	248518	249327	269	tail sheath stabilizer and completion protein
ORF369	-	249342	251126	594	Hypothetical protein
ORF370	-	251158	255111	1317	Hypothetical protein
ORF371	-	255134	258442	1102	Hypothetical protein
ORF372	-	258445	261711	1088	Hypothetical protein
ORF373	-	261719	263035	438	Hypothetical protein
ORF374	-	263032	263712	226	Hypothetical protein
ORF375	-	263709	264341	210	Hypothetical protein
ORF376	+	264390	264911	173	Predicted ORF
ORF377	+	265015	267459	814	ribonucleotide-diphosphate reductase alpha subunit
ORF378	+	267547	268599	350	ribonucleotide-diphosphate reductase beta subunit
ORF379	+	268660	269532	290	Hypothetical protein
ORF380	+	269560	270006	148	Predicted ORF
ORF381	+	270006	270368	120	Predicted ORF
ORF382	-	270523	271203	226	Papain family cysteine protease
ORF383	+	271255	272136	293	metallophosphoesterase
ORF384	+	272200	280671	2823	glycerophosphoryl diester phosphodiesterase
ORF385	-	280687	281289	200	Predicted ORF
ORF386	-	281324	281746	140	Predicted ORF
ORF387	-	281811	282134	107	Predicted ORF
ORF388	+	282226	282804	192	SAM-dependent methyltransferase
ORF389	+	282835	283050	71	Predicted ORF
ORF390	-	283076	283423	115	L,D-transpeptidase
ORF391	-	283498	285087	529	Hypothetical protein
ORF392	-	285246	286136	296	thymidylate synthase
ORF393	-	286133	286327	64	Predicted ORF
ORF394	-	286336	286626	96	DUF4326 domain containing protein
ORF395	-	286623	286796	57	Predicted ORF
ORF396	-	286828	288915	695	Hypothetical protein
ORF397	-	289411	289941	176	Hypothetical protein
ORF398	-	289931	290326	131	Predicted ORF
ORF399	-	290323	291186	287	nucleotidyltransferase
ORF400	-	291229	291489	86	Predicted ORF
ORF401	-	291486	292103	205	Hypothetical protein
ORF402	-	292103	293038	311	Predicted ORF
ORF403	-	293039	294073	344	GMP reductase
ORF404	-	294073	294279	68	Predicted ORF
ORF405	-	294321	294728	135	Predicted ORF
ORF406	-	294773	295942	389	ATPase
ORF407	-	296069	296296	75	Predicted ORF
ORF408	-	296355	296621	88	Predicted ORF
ORF409	-	296618	296986	122	acyl-CoA thioesterase
ORF410	-	296983	297201	72	Predicted ORF
ORF411	-	297262	297648	128	Predicted ORF
ORF412	-	297703	298032	109	Hypothetical protein
ORF413	-	298048	298698	216	von Willebrand factor type A domain containing protein
ORF414	-	298708	299979	423	poly (ADP-ribose) polymerase
ORF415	-	300042	300296	84	Hypothetical protein
ORF416	-	300337	300726	129	Predicted ORF
ORF417	-	300726	301010	94	Predicted ORF
ORF418	-	301007	301258	83	Hypothetical protein
ORF419	-	301298	301750	150	Hypothetical protein
ORF420	-	301743	302558	271	DUF4343 domain containing protein
ORF421	-	302555	302857	100	Predicted ORF
ORF422	-	302844	303419	191	Hypothetical protein
ORF423	-	303453	303932	159	M23 family peptidase
ORF424	-	303929	304177	82	Predicted ORF
ORF425	-	304178	304417	79	Predicted ORF
ORF426	-	304471	304857	128	Predicted ORF
ORF427	-	304995	305222	75	Predicted ORF
ORF428	-	305256	305636	126	Hypothetical protein
ORF429	+	305767	305961	64	Predicted ORF

ORF430	-	305991	306386	131	Predicted ORF
ORF431	-	306418	306753	111	Predicted ORF
ORF432	-	306771	307415	214	ABC transporter ATP-binding protein
ORF433	-	307424	307876	150	MazG nucleotide pyrophosphohydrolase
ORF434	-	307942	308400	152	Predicted ORF
ORF435	-	308407	309087	226	Predicted ORF
ORF436	-	309080	309487	135	Predicted ORF
ORF437	-	309471	309926	151	Predicted ORF
ORF438	-	309931	310449	172	Predicted ORF
ORF439	-	310484	310990	168	Predicted ORF
ORF440	-	311002	311325	107	Predicted ORF
ORF441	-	311382	311699	105	Hypothetical protein
ORF442	-	311696	312010	104	Predicted ORF
ORF443	-	312148	312390	80	Predicted ORF
ORF444	-	312393	312584	63	Predicted ORF
ORF445	-	312806	313030	74	Hypothetical protein
ORF446	-	313058	314116	352	CCA tRNA nucleotidyl transferase
ORF447	-	314169	314630	153	Predicted ORF
ORF448	-	314633	315169	178	Predicted ORF
ORF449	+	315202	315636	144	Predicted ORF
ORF450	+	315633	316253	206	Predicted ORF
ORF451	+	316250	316474	74	Hypothetical protein
ORF452	+	316471	316674	67	Predicted ORF
ORF453	+	316671	316838	55	Predicted ORF
ORF454	+	316835	317002	55	Predicted ORF
ORF455	+	316995	317330	111	Predicted ORF
ORF456	+	317327	317503	58	Predicted ORF
ORF457	+	317529	317870	113	Hypothetical protein
ORF458	-	317907	318209	100	Predicted ORF
ORF459	-	318310	318633	107	Predicted ORF
ORF460	-	318887	319120	77	Predicted ORF
ORF461	-	319176	319538	120	Predicted ORF
ORF462	-	319624	320013	129	Hypothetical protein
ORF463	-	320022	320204	60	Predicted ORF
ORF464	-	320264	320527	87	Hypothetical protein
ORF465	-	320650	320841	63	Predicted ORF
ORF466	-	320841	320966	41	Predicted ORF
ORF467	+	321035	321256	73	Predicted ORF
ORF468	-	321266	321655	129	Hypothetical protein
ORF469	-	321694	321981	95	Predicted ORF
ORF470	-	322133	322369	78	Predicted ORF
ORF471	-	322371	323045	224	Predicted ORF
ORF472	-	323098	323499	133	Predicted ORF
ORF473	-	323552	323950	132	Hypothetical protein
ORF474	-	324143	324442	99	Predicted ORF
ORF475	-	324490	324834	114	Predicted ORF
ORF476	-	324836	325039	67	Predicted ORF
ORF477	-	325091	325309	72	Predicted ORF
ORF478	-	325306	325473	55	Predicted ORF
ORF479	-	325511	325804	97	Hypothetical protein
ORF480	-	325801	325998	65	Predicted ORF
ORF481	-	325998	326324	108	Predicted ORF
ORF482	-	326355	326468	37	Predicted ORF
ORF483	-	326528	326758	76	Predicted ORF
ORF484	-	326953	327183	76	Predicted ORF
ORF485	-	327186	327344	52	Predicted ORF
ORF486	-	327416	327634	72	Predicted ORF
ORF487	-	328202	328435	77	Predicted ORF
ORF488	-	328484	328804	106	Hypothetical protein
ORF489	-	328804	328983	59	Hypothetical protein
ORF490	-	328983	329219	78	Hypothetical protein
ORF491	-	329216	329356	46	Predicted ORF
ORF492	-	329411	330019	202	Hypothetical protein
ORF493	-	330231	330506	91	Predicted ORF
ORF494	-	330557	330664	35	Predicted ORF
ORF495	-	330775	330966	63	Predicted ORF
ORF496	-	331034	331264	76	Predicted ORF
ORF497	-	331266	331550	94	Hypothetical protein
ORF498	-	331712	332215	167	Hypothetical protein
ORF499	-	332370	332585	71	Predicted ORF
ORF500	-	332706	333449	247	Predicted ORF
ORF501	-	333526	333726	66	Predicted ORF

ORF502	-	333967	334299	110	Hypothetical protein
ORF503	-	334361	334726	121	Predicted ORF
ORF504	-	334791	335306	171	SprT-like protein
ORF505	-	335367	335618	83	Predicted ORF
ORF506	-	335783	336049	88	Predicted ORF
ORF507	-	336245	336490	81	Predicted ORF
ORF508	-	336503	336667	54	Predicted ORF
ORF509	-	336727	337134	135	Predicted ORF
ORF510	-	337131	337361	76	Hypothetical protein
ORF511	-	337532	337789	85	Predicted ORF
ORF512	-	337906	338085	59	Predicted ORF
ORF513	-	338093	338434	113	Hypothetical protein
ORF514	-	338453	338650	65	Predicted ORF
ORF515	-	338659	338967	102	Predicted ORF
ORF516	-	339034	339246	70	Predicted ORF
ORF517	+	339355	339762	135	Hypothetical protein
ORF518	-	339759	340061	100	Hypothetical protein
ORF519	-	340121	340345	74	Predicted ORF
ORF520	-	340342	340506	54	Predicted ORF
ORF521	-	340539	340919	126	HNH endonuclease
ORF522	-	340930	341178	82	Predicted ORF
ORF523	+	341354	341803	149	N-acetyltransferase
ORF524	+	342052	342573	173	Predicted ORF
ORF525	+	342622	342870	82	Hypothetical protein
ORF526	+	342870	343106	78	Predicted ORF
ORF527	+	343146	343499	117	Predicted ORF
ORF528	+	343617	343982	121	Predicted ORF
ORF529	+	344110	344862	250	Hypothetical protein
ORF530	+	344911	345417	168	Hypothetical protein
ORF531	+	345427	345606	59	Predicted ORF
ORF532	+	345664	346101	145	Predicted ORF
ORF533	+	346101	346682	193	Hypothetical protein
ORF534	+	346684	346983	99	Predicted ORF
ORF535	+	347018	347422	134	Predicted ORF
ORF536	+	347432	348070	212	GTP pyrophosphokinase
ORF537	+	348076	348729	217	Predicted ORF
ORF538	+	348840	349064	74	Predicted ORF
ORF539	+	349910	350821	303	Band 7 protein
ORF540	+	350929	351348	139	Predicted ORF
ORF541	+	351433	353094	553	Hypothetical protein
ORF542	+	353112	353468	118	Predicted ORF
ORF543	+	353465	353707	80	Predicted ORF
ORF544	+	353740	353934	64	Predicted ORF
ORF545	+	353975	354286	103	Predicted ORF
ORF546	+	354296	354835	179	Hypothetical protein
ORF547	+	355041	355355	104	Hypothetical protein
ORF548	+	358641	359006	121	Predicted ORF
ORF549	+	359057	359335	92	Predicted ORF
ORF550	+	359680	360408	242	Predicted ORF
ORF551	+	360405	360608	67	Predicted ORF
ORF552	+	360766	360993	75	Hypothetical protein
ORF553	+	360980	361114	44	Predicted ORF
ORF554	+	361490	361747	85	Predicted ORF
ORF555	+	361819	362094	91	Predicted ORF
ORF556	+	362215	362421	68	Predicted ORF
ORF557	+	362651	362899	82	Predicted ORF
ORF558	+	363007	363396	129	Predicted ORF
ORF559	+	363406	363777	123	Predicted ORF
ORF560	+	364262	364531	89	Predicted ORF
ORF561	+	364552	364761	69	Predicted ORF
ORF562	+	365500	365910	136	Predicted ORF
ORF563	+	365910	366191	93	Predicted ORF
ORF564	+	366417	366575	52	Predicted ORF
ORF565	+	366739	367425	228	Predicted ORF
ORF566	+	367843	368055	70	Predicted ORF
ORF567	+	368381	368623	80	Predicted ORF
ORF568	+	368835	369215	126	Predicted ORF
ORF569	+	369396	370010	204	Predicted ORF
ORF570	+	370035	370397	120	Predicted ORF
ORF571	+	370486	371085	199	cell wall hydrolase
ORF572	+	371090	371704	204	Predicted ORF
ORF573	+	371769	372194	141	Predicted ORF

ORF574	+	372291	372488	65	Predicted ORF
ORF575	+	372520	372720	66	Predicted ORF
ORF576	-	372722	373549	275	Predicted ORF
ORF577	-	373661	374395	244	M23 family peptidase
ORF578	-	374514	375362	282	Hypothetical protein
ORF579	-	375359	375574	71	Predicted ORF
ORF580	-	375571	376878	435	RNA ligase
ORF581	-	376992	377993	333	protein rIIB
ORF582	-	378059	380266	735	protein rIIA
ORF583	-	380538	380699	53	Predicted ORF
ORF584	-	380761	381111	116	Predicted ORF
ORF585	-	381098	381847	249	$tRNA^{His}$ guanylyltransferase
ORF586	-	381847	382383	178	Predicted ORF
ORF587	-	382383	382646	87	Predicted ORF
ORF588	-	382702	382923	73	Predicted ORF
ORF589	-	382958	383305	115	Predicted ORF
ORF590	-	383302	383478	58	Predicted ORF
ORF591	-	383511	383819	102	Predicted ORF
ORF592	-	383842	384186	114	Predicted ORF

Supplementary Table S3. Annotation of XacN1 tRNA genes.

Amino acid	Anticodon	Number of tRNA
Ala	TGC	2
Arg	TCT	2
Asn	GTT	2
Asp	GTC	2
Cys	GCA	2
Gln	CTG	2
	TTG	2
Glu	TTC	2
Gly	TCC	2
His	GTG	2
Ile	GAT	2
Leu	CAA	2
	TAG	2
Lys	CTT	2
	TTT	2
Met	CAT	4
Phe	GAA	2
Pro	TGG	4
Ser	GCT	2
	TGA	2
Thr	TGT	2
Trp	CCA	2
Tyr	GTA	2
Val	TAC	2
Suppressor	CTA	4
Pseudogene	TTC	2

Supplementary Table S4. Comparison of codon frequency and number of tRNA gene in XacN1 and *X. citri* genome.

	Anti codon	XacN1 frequency	*X. citri* frequency	Ratio (phage/host)	XacN1 tRNA	*X. citri* tRNA
Phage<Host	CGC	0.0127	0.0500	0.25	0	1
	CCG	0.0028	0.0105	0.26	0	1
	CAG	0.0222	0.0680	0.33	0	2
	CCC	0.0035	0.0100	0.35	0	1
	GGG	0.0049	0.0119	0.41	0	1
	GCC	0.0249	0.0579	0.43	0	2
	GGC	0.0242	0.0561	0.43	0	2
	CAC	0.0200	0.0450	0.44	0	1
	GCT	0.0132	0.0222	0.59	2	1
	GAG	0.0085	0.0141	0.60	0	1
	CGG	0.0223	0.0325	0.69	0	1
	CTG	0.0251	0.0362	0.69	2	1
	TAA	0.0006	0.0008	0.72	0	1
	CTC	0.0170	0.0235	0.72	0	1
	CAA	0.0141	0.0192	0.73	2	1
	GCA	0.0056	0.0072	0.78	2	1
	CCT	0.0012	0.0015	0.81	0	1
	GGT	0.0263	0.0323	0.81	0	1
	CGA	0.0142	0.0170	0.83	0	1
	GTG	0.0114	0.0131	0.87	2	1
	TGC	0.0152	0.0173	0.88	2	3
Phage=Host	GAC	0.0199	0.0214	0.93	0	1
	GAT	0.0323	0.0341	0.95	2	2
	TTG	0.0087	0.0091	0.96	2	1
	CCA	0.0152	0.0157	0.96	2	1
	GGA	0.0101	0.0096	1.05	0	1
	CGT	0.0154	0.0145	1.06	0	1
Phage>Host	GTC	0.0407	0.0350	1.16	2	2
	CAT	0.0254	0.0206	1.23	4	3
	GAA	0.0358	0.0263	1.36	2	1
	GTT	0.0315	0.0188	1.68	2	1
	GTA	0.0286	0.0160	1.79	2	1
	TCC	0.0075	0.0040	1.90	2	1
	TTC	0.0478	0.0244	1.96	4	2
	TTT	0.0090	0.0046	1.97	2	1
	ACG	0.0227	0.0114	2.00	0	2
	CTT	0.0534	0.0219	2.44	2	1
	TGG	0.0110	0.0044	2.51	4	1
	TAC	0.0099	0.0032	3.09	2	1
	TCT	0.0018	0.0006	3.24	2	1
	TGT	0.0113	0.0024	4.64	2	1
	TGA	0.0089	0.0013	7.08	2	1
	TAG	0.0195	0.0020	9.88	2	1

Supplementary Table S5. XacN1 ORFs classified in major functional categories.

Function	ORF	Encoded phage protein	Related virus or bacteria	Similarity (%)
Structual proteins	274	major capsid	*Cronobacter* phage vB_CsaM_GAP32	37.3
	301	portal vertex of head	*Cronobacter* phage vB_CsaM_GAP32	36.7
	317	baseplate hub	*Enterobacteria* phage vB_KleM-RaK2	22.2
	323	baseplate wedge	*Aeromonas* phage PX29	37.9
	324	baseplate wedge	*Cronobacter* phage vB_CsaM_GAP32	36.5
	348	head completion	*Pelagibacter* phage HTVC008M	44.2
	351	structural protein	*Escherichia* phage 121Q	29.4
	367	tail sheath	*Escherichia* phage 121Q	38.3
	368	tail sheath stabilizer and completion	*Cronobacter* phage vB_CsaM_GAP32	29.2
DNA replication	176	DNA primase	*Methylobacterium gossipiicola*	27.9
	178	DNA ligase	*Acanthamoeba polyphaga moumouvirus*	34.3
	181	DNA topoisomerase II large subunit	*Deftia* phage phiW-14	45.3
	182	DNA topoisomerase II medium subunit	*Serratia* phage phiMAM1	30.9
	223	DNA polymerase III epsilon subunit	*Desulfosporosinus orientis*	29.3
	241	sliding clamp loader	*Cronobacter* phage vB_CsaM_GAP32	34.1
	245	DNA polymerase III alpha subunit	*Escherichia* phage 121Q	54.7
	248	DNA helicase	*Escherichia* phage 121Q	34.4
	252	single-stranded DNA binding protein	*Enterobacteria* phage vB_KleM-RaK2	25.7
	266	DNA primase/helicase	*Escherichia* phage 121Q	30.6
	340	RNase H	*Enterobacteria* phage vB_KleM-RaK2	32.0
	343	DNA polymerase	*Enterobacteria* phage vB_KleM-RaK2	27.1
DNA recombination and	200	alkylated DNA repair dioxygenase	*Methylibium petroleiphilum*	47.0
	251	RecA-like protein	*Cronobacter* phage vB_CsaM_GAP32	39.1
	287	NUDIX hydrolase	*Xanthomonas* phage Xp15	36.4
	305	recombination endonuclease subunit	*Escherichia* phage 121Q	28.0
	312	Rad3-related DNA helicase	*Truepera radiovictrix*	30.0
Nucleotide metabolism	186	dihydrofolate reductase	*Massilia* sp. Leaf139	39.5
	204	deoxycytidylate deaminase	*Acidobacteriaceae bacterium* URHE0068	49.0
	224	deoxynucleoside monophosphate kinase	*Marinobacterium stanieri*	38.2
	377	ribonucleotide-diphosphate reductase alpha subunit	*Escherichia* phage 121Q	49.1
	378	ribonucleotide-diphosphate reductase beta subunit	*Marinomonas posidonica*	48.5
	392	thymidylate synthase	*Cryptosporidium muris* RN66	42.5
	403	GMP reductase	*Azospirillum brasilense*	50.4

Supplementary Table S6. Orthologous genes conserved in XacN1 and one or more phages in the monophyletic group but not found in *Escherichia* phage T4.

Annotation	XacN1 ORF	SCTP-2	Atu_ph07	BF	CBB	GAP32	PBECO4	RaK2	121Q	slurp01	K64-1
Hypothetical protein	275	x[a]	-	x	x	x	x	x	x	x	x
Hypothetical protein	349	x	-	x	x	x	x	x	x	x	x
PhoH	188	-	-	x	x	x	x	x	x	x	x
DNA polymerase III alpha subunit	245	x	-	x	x	x	x	x	x	x	-
HNH endonuclease	521, 62	x	-	x	x	x	x	x	x	x	-
Hypothetical protein	119, 578	-	-	x	x	x	x	x	x	x	x
CCA tRNA nucleotidyl transferase	446	-	-	x	x	x	x	x	x	x	x
Hypothetical protein	259	-	-	x	x	x	x	x	x	x	x
Hypothetical protein	243	x	-	x	x	x	x	x	x	x	-
structural protein	351	x	-	x	x	x	x	x	x	x	-
Hypothetical protein	20, 479	x	-	x	x	x	x	x	x	x	-
Hypothetical protein	235, 236, 237	-	-	x	x	x	x	x	x	x	x
Hypothetical protein	228	x	-	x	x	x	x	x	x	x	-
Hypothetical protein	239	x	-	x	x	x	x	x	x	x	-
Hypothetical protein	247	x	-	x	x	x	x	x	x	x	-
Hypothetical protein	238	x	-	x	x	x	x	x	x	x	-
Hypothetical protein	286	-	-	x	x	x	x	x	x	x	-
Clp protease	177	-	-	x	x	x	x	x	x	x	-
Hypothetical protein	258	-	-	x	x	x	x	x	x	x	-
Hypothetical protein	249	-	-	x	x	x	x	x	x	x	-
Hypothetical protein	401	-	-	x	x	x	x	x	x	x	-
co-chaperone GroES	282	-	-	x	x	x	x	x	x	x	-
metallophosphoesterase	383	x	-	x	x	x	x	-	x	x	-
Hypothetical protein	106, 565	-	-	x	x	x	x	x	x	x	-
Hypothetical protein	329	-	-	x	x	x	x	x	x	x	-
nicotinate phosphoribosyltransferase	206	x	-	x	x	x	-	x	-	-	x
Hypothetical protein	23, 482	x	-	x	x	x	-	-	x	x	-
bifunctional nicotinamide mononucleotide adenylyltransferase/ADP-ribose	216	x	-	x	x	x	-	x	-	-	x
Hypothetical protein	133, 592	-	-	x	x	x	x	-	x	x	-
Hypothetical protein	341	-	-	x	-	x	x	x	x	x	-
Hypothetical protein	117, 576	-	-	x	x	x	x	-	x	x	-
Hypothetical protein	318	x	x	-	-	x	x	x	x	-	-
Hypothetical protein	257	-	-	x	x	x	-	x	-	x	-
GTP pyrophosphokinase	536, 77	x	-	x	x	x	-	x	-	-	-
peptidyl-tRNA hydrolase	295	x	-	x	x	x	-	x	-	-	-
Hypothetical protein	319	-	x	-	-	x	x	x	x	-	-
Hypothetical protein	267	-	-	-	-	x	x	x	x	-	x
Hypothetical protein	226	-	x	-	-	x	x	x	x	-	-
phosphoesterase	293	x	-	x	x	x	-	-	-	-	-
Hypothetical protein	364	-	-	x	x	x	-	x	-	-	-
glutamyl-tRNA amidotransferase	360	x	-	x	x	x	-	-	-	-	-
tRNA^{His} guanylyltransferase	126, 585	x	-	x	x	x	-	-	-	-	-
DUF4343 domain containing protein	420	x	-	x	x	x	-	-	-	-	-
Hypothetical protein	426	-	-	-	-	-	x	x	x	x	-
Hypothetical protein	170	-	-	-	-	-	x	x	x	x	-
Hypothetical protein	208	x	-	x	x	x	-	-	-	-	-
Band 7 protein	539, 80	-	-	x	x	x	-	-	-	-	-
RNA ligase	121, 580	-	-	x	x	x	-	-	-	-	-
ATPase	406	x	-	-	x	x	-	-	-	-	-
Hypothetical protein	537, 78	-	-	x	x	x	-	-	-	-	-
Hypothetical protein	441	-	-	x	x	x	-	-	-	-	-
Hypothetical protein	357	-	-	x	x	x	-	-	-	-	-
methyltransferase	202	-	-	x	x	x	-	-	-	-	-
Hypothetical protein	203	-	-	-	-	-	x	-	x	x	-
Hypothetical protein	141	-	-	-	x	x	-	-	-	-	-
DUF1768 domain containing protein	213, 289	-	-	x	x	-	-	-	-	-	-
SprT-like protein	45, 504	x	-	-	-	x	-	-	-	-	-
Hypothetical protein	308	-	-	-	x	x	-	-	-	-	-
Hypothetical protein	459	-	-	-	x	x	-	-	-	-	-
Hypothetical protein	350	-	-	-	-	x	-	-	-	-	-
Hypothetical protein	232	-	-	-	-	-	-	x	-	-	-
Hypothetical protein	435	-	x	-	-	-	-	-	-	-	-
M23 family peptidase	118, 302, 322, 423, 577	-	x	-	-	-	-	-	-	-	-
Hypothetical protein	43, 502	-	-	-	-	-	-	x	-	-	-
Hypothetical protein	29, 488	x	-	-	-	-	-	-	-	-	-

Hypothetical protein	335	-	-	-	-	x	-	-	-	-	-
5' nucleotidase	172	-	x	-	-	-	-	-	-	-	-
predicted kinase	199	x	-	-	-	-	-	-	-	-	-
Hypothetical protein	24, 376, 483	x	-	-	-	-	-	-	-	-	-
Hypothetical protein	339	x	-	-	-	-	-	-	-	-	-
putative NUDIX hydrolase	287	x	-	-	-	-	-	-	-	-	-
alkylated DNA repair dioxygenase	200	x	-	-	-	-	-	-	-	-	-
Hypothetical protein	396	x	-	-	-	-	-	-	-	-	-

[a] 'x' indicates the presence, while '-' indicates the absence.

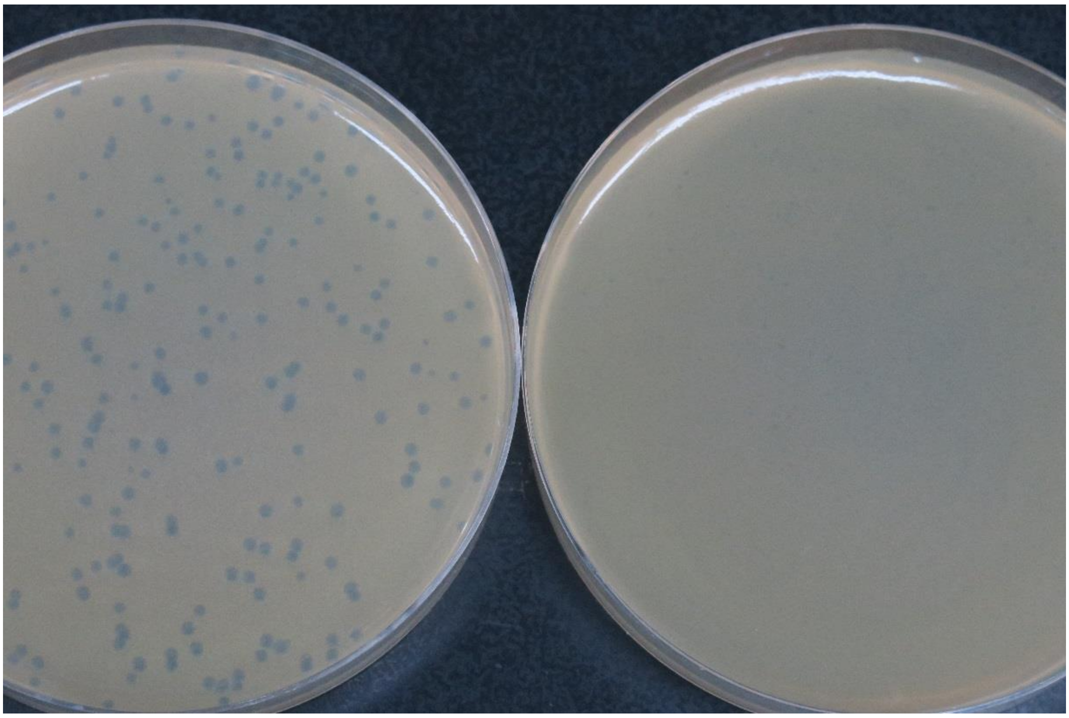

Supplementary Fig. S1. Plaques of XacN1 formed with *X. citri* MAFF 301080 as the host. The phage formed clear plaques (1-2 mm) on 0.3% top agar (left panel), but formed very small plaques when the top agar concentration was increased to 0.45% (right panel).

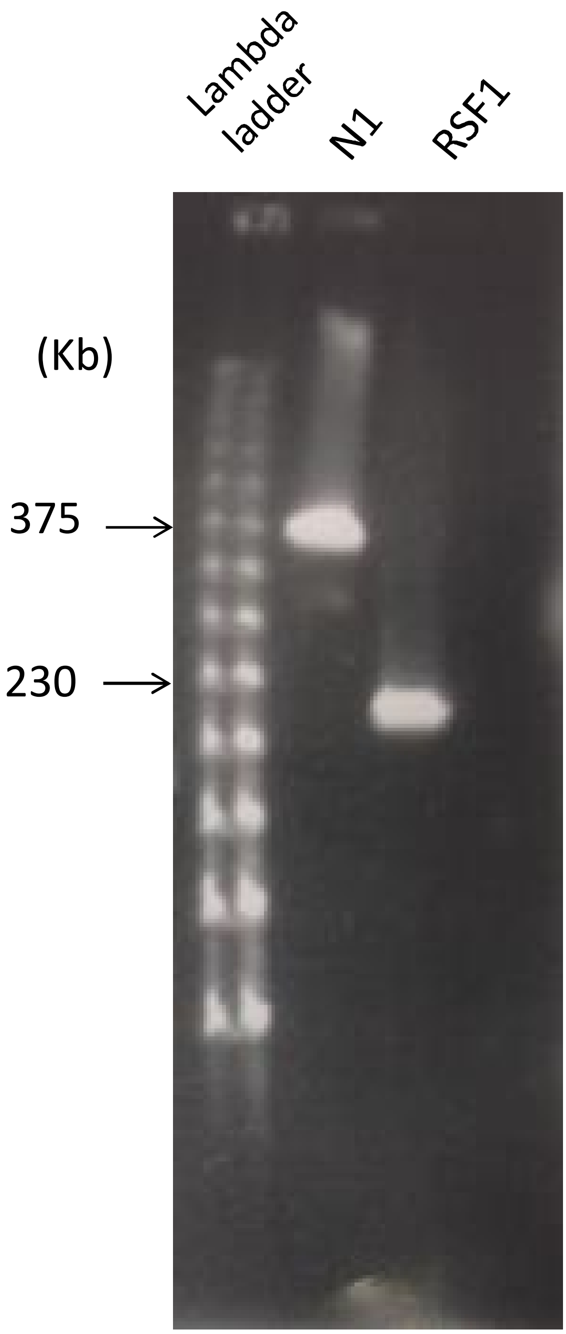

Supplementary Fig. S2. Pulsed-field gel electrophoresis of phage DNA. Purified phage particles embedded in agarose were treated with proteinase K and Sarkosyl, and subjected to CHEF Mapper analysis. Lanes: 1, λ ladder (size marker); 2, XacN1; 3, RSF1 (223 kbp, Bhunchoth *et al.*, 2016).

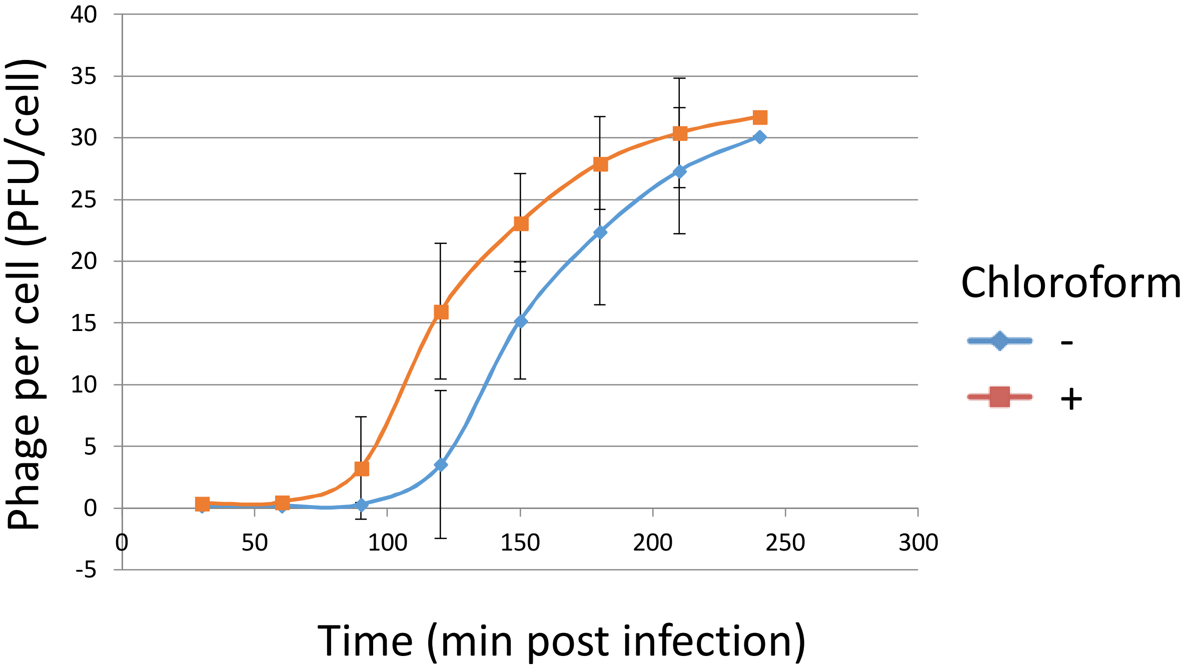

Supplementary Fig. S3. Single-step growth curve of XacN1 growing on *X. citri* MAFF 301080. Shown are the PFUs per infected cell in cultures at different time post infection. Samples were taken at 30-min intervals and immediately diluted with or without chloroform treatment, and the titers were determined by the double layered agar plate method. The eclipse period and the burst size were 60 min and 30 PFU/cell, respectively. Experiments were repeated six times.

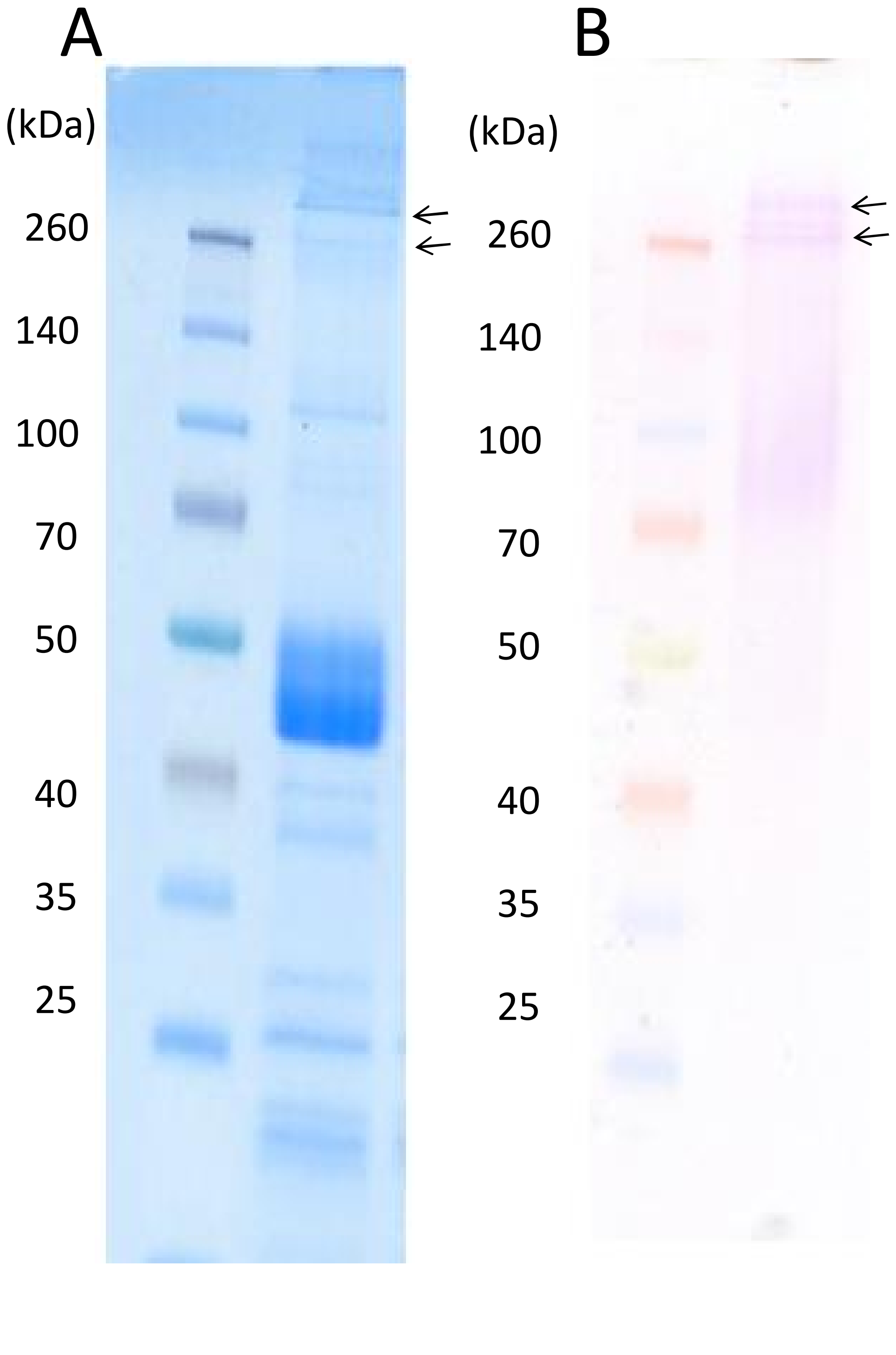

Supplementary Fig. S4. Detection of glycoproteins in XacN1 virions. Virion proteins separated by SDS-polyacryl amide gel electrophoresis were stained with Coomassie brilliant blue (A) and with Schiffôs reagent (using a Pierce glycoprotein staining kit, Thermo Fisher Scientific Inc., Waltham, MA, USA) (B). Arrows indicate stained bands. Molecular markers are a standard ladder of Spectra Multicolor Broad Range (Thermo Fisher Scientific Inc.)

A

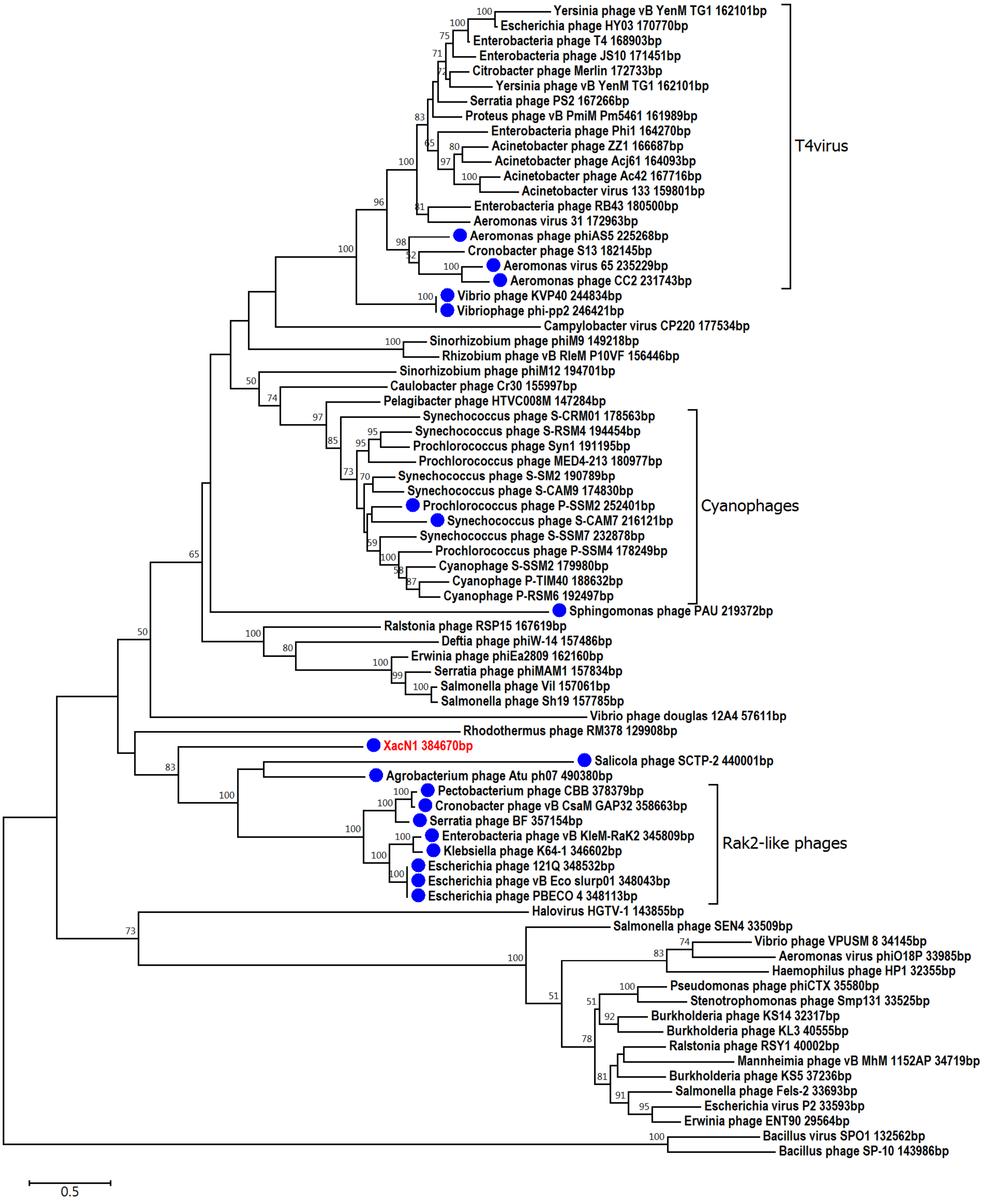

Supplementary Fig. S5. Maximum likelihood phylogenetic trees of (A) Terminase large subunit proteins, (B) Major capsid proteins, and (C) Tail sheath proteins. Blue dots represent genome size 200 kbp and more.

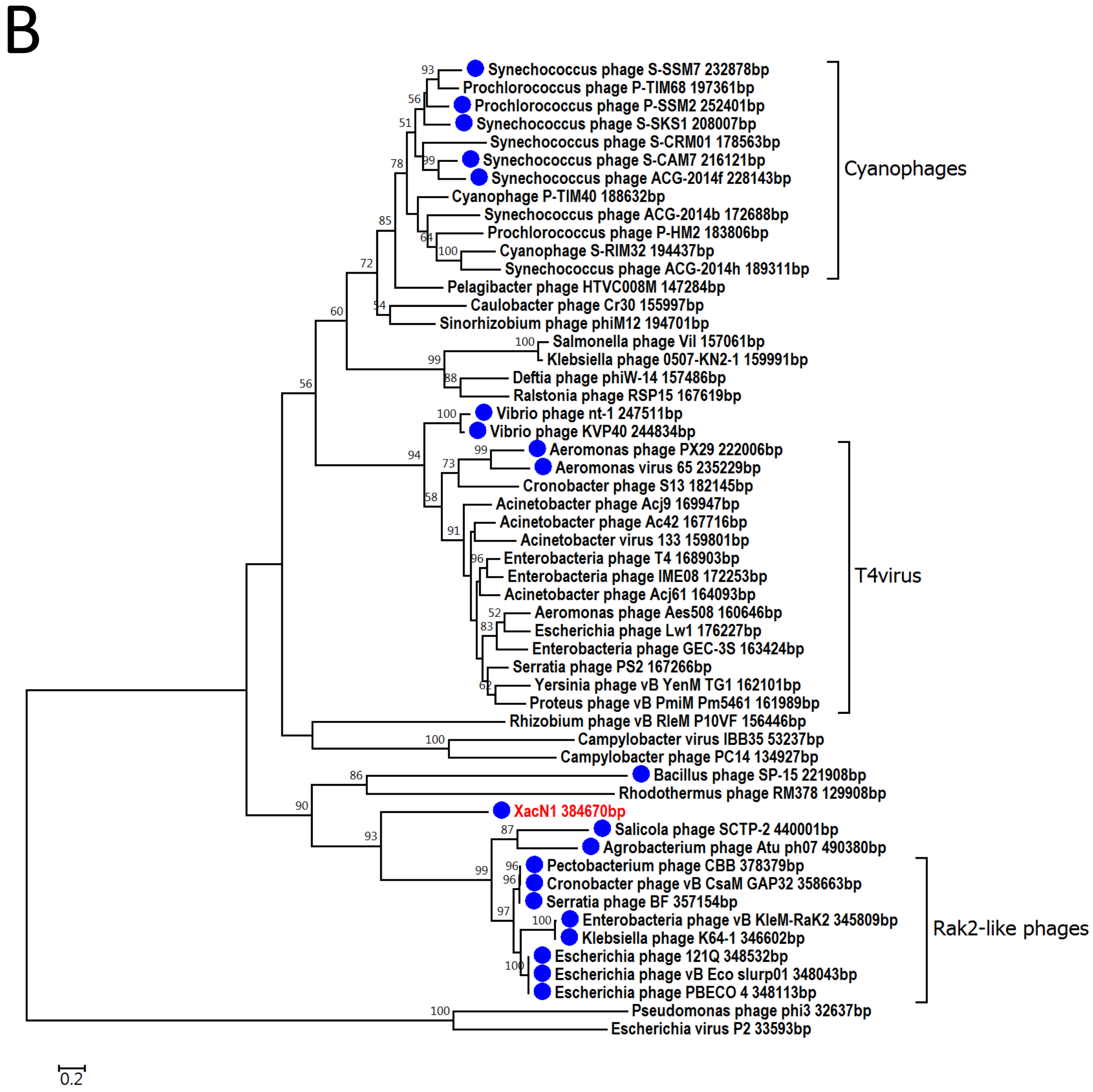

Supplementary Fig. S5.

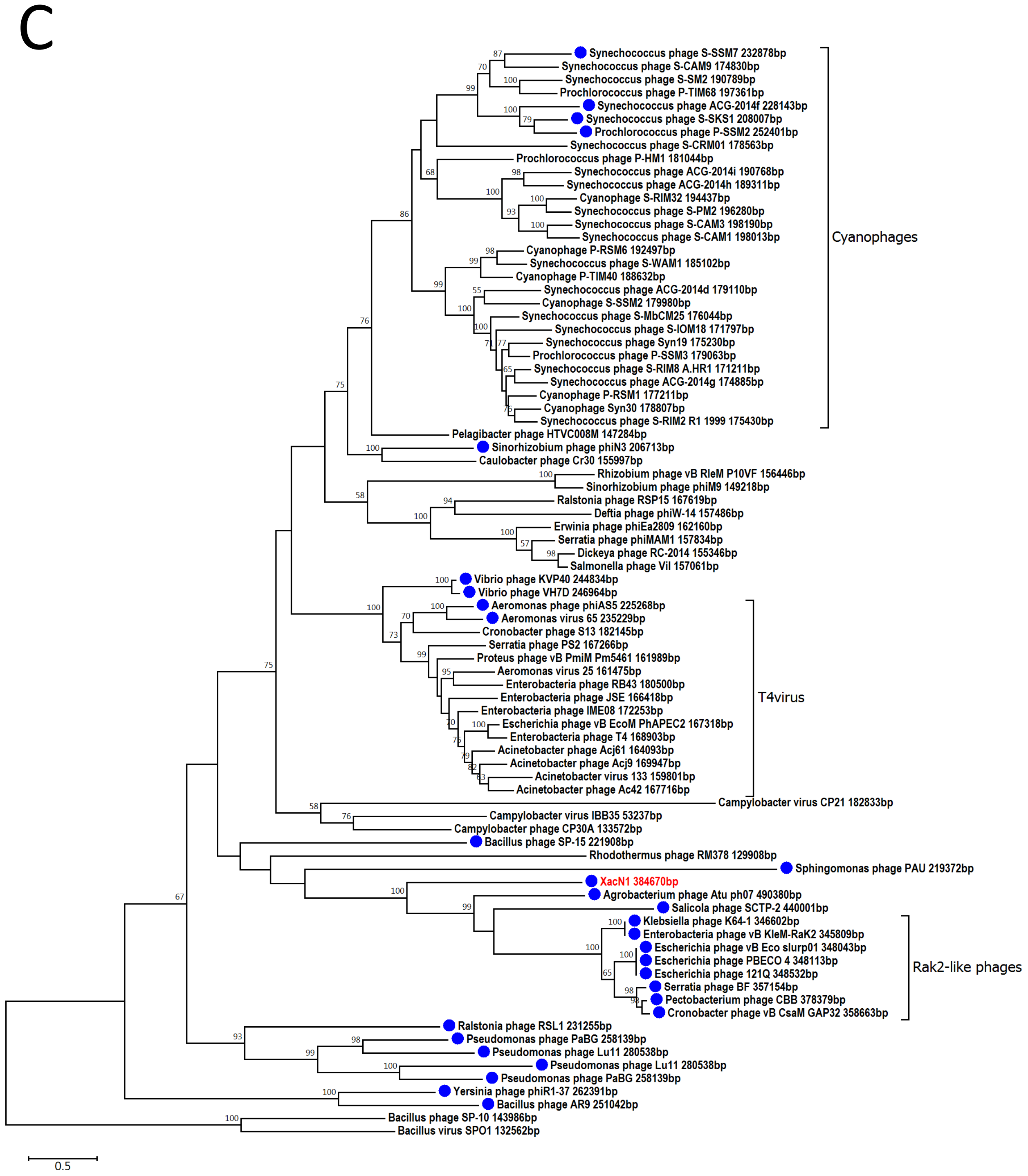

Supplementary Fig. S5.

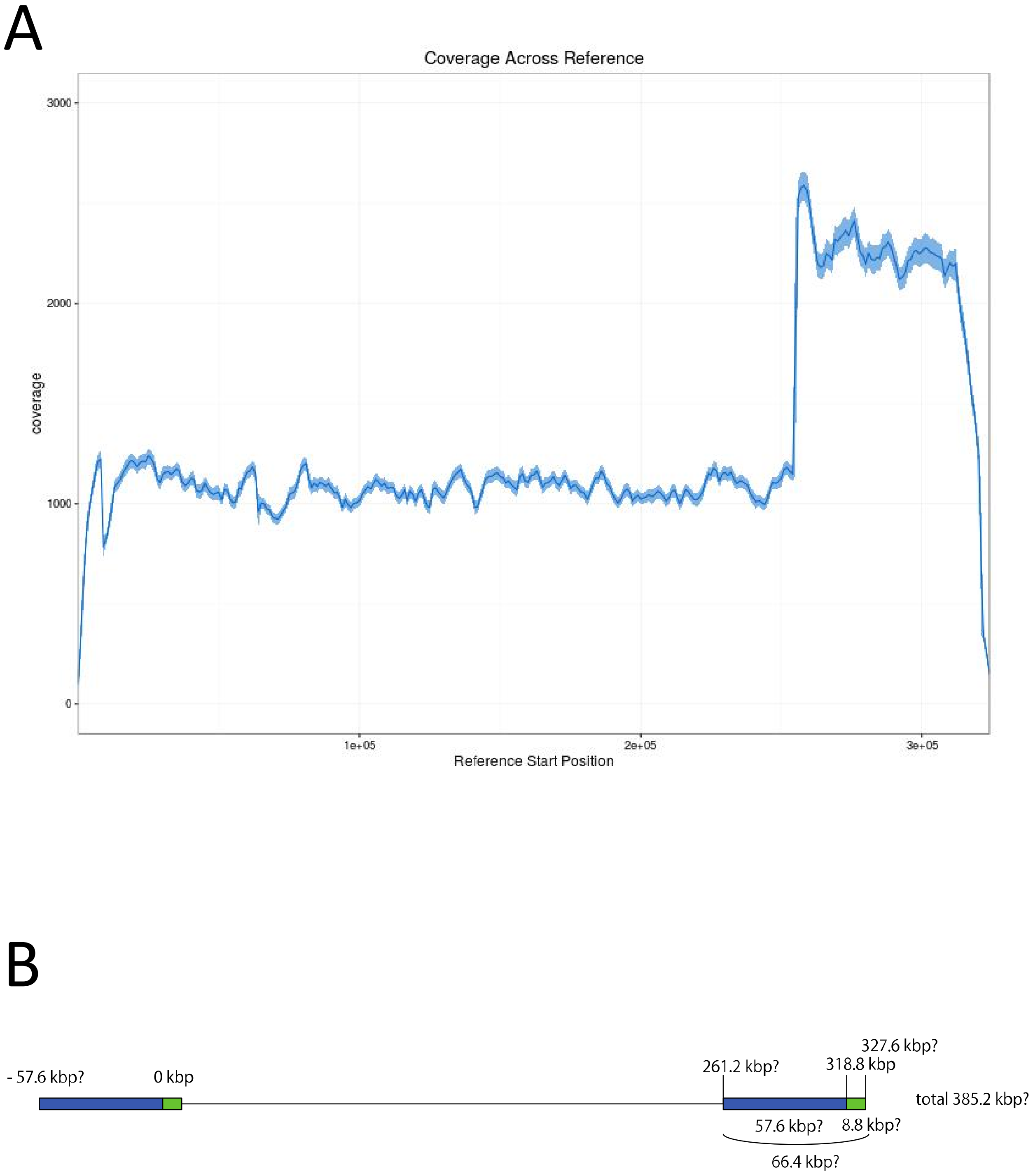

Supplementary Fig. S6. Evidence and sequence determination of direct terminal repeats of XacN1 genome. (A) Coverage of the assembled sequences of XacN1 DNA. The 70 kbp terminal region (right extremity) showed double the coverage compared with the rest, indicating a duplication of this region on the genome. (B) A model of XacN1 genomic DNA where a 70-kbp region repeated on both ends of the genome.

C

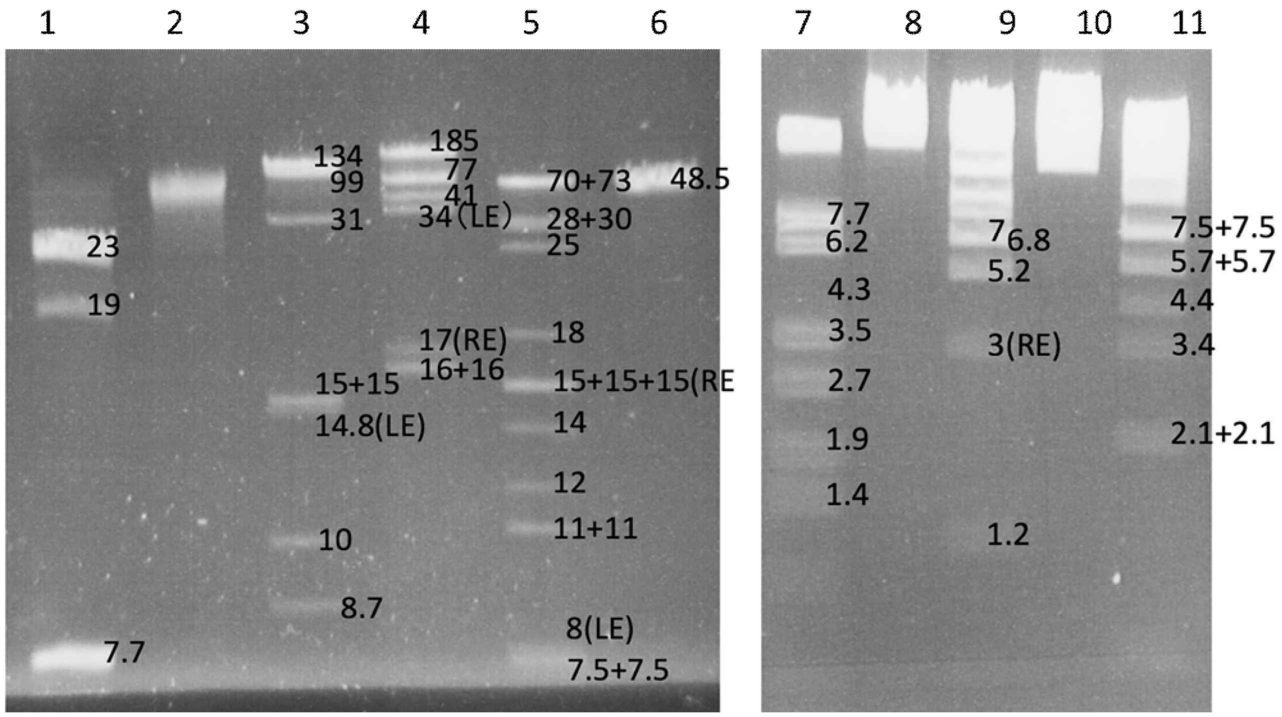

Supplementary Fig. S6. (C) Restriction enzyme digestion patterns of the XacN1 genomic DNA. Lanes: 1, Lamda DNA *Sty*I fragments; 2, *Bst*HKAI; 3, *Bss*HII; 4, *Nae*I; 5, *Spe*I; 6, Lambda DNA; 7, Lambda DNA *Sty*I fragments; 8, *Bst*HKAI; 9, *Bss*HII; 10, *Nae*I; 11, *Spe*I. DNA fragments in lanes 1-6 were separated under an electrophoresis condition to see higher molecules, while those in lanes 7-11 were for lower molecules.

D

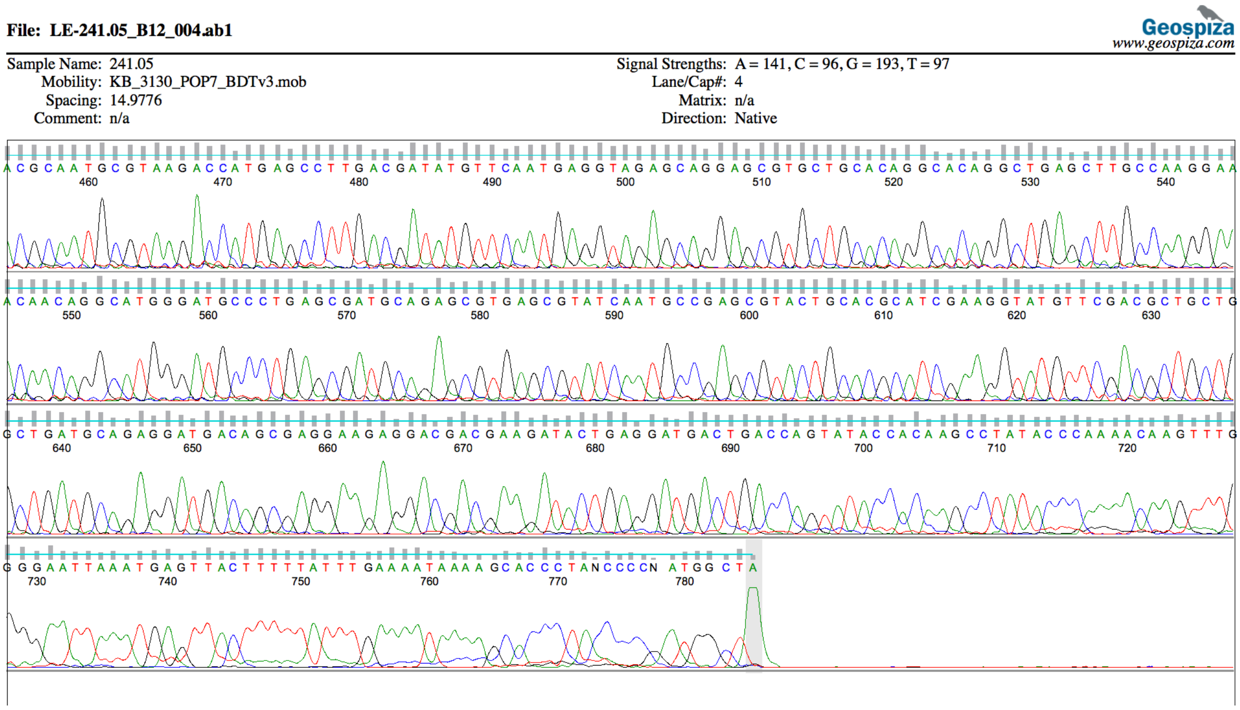

Supplementary Fig. S6. (D) Direct sequencing of the left end of XacN1 DNA. The primer used is TTA GGG GTT GAC ATT TGT CAG CCC CTT TTG, corresponding to XacN1 positions 781-810 (reversed).

E

File: 411.01_A11_001.ab1

Sample Name:	411.01	Signal Strengths:	A = 13, C = 11, G = 14, T = 13
Mobility:	KB_3130_POP7_BDTv3.mob	Lane/Cap#:	1
Spacing:	12.7947	Matrix:	n/a
Comment:	n/a	Direction:	Native

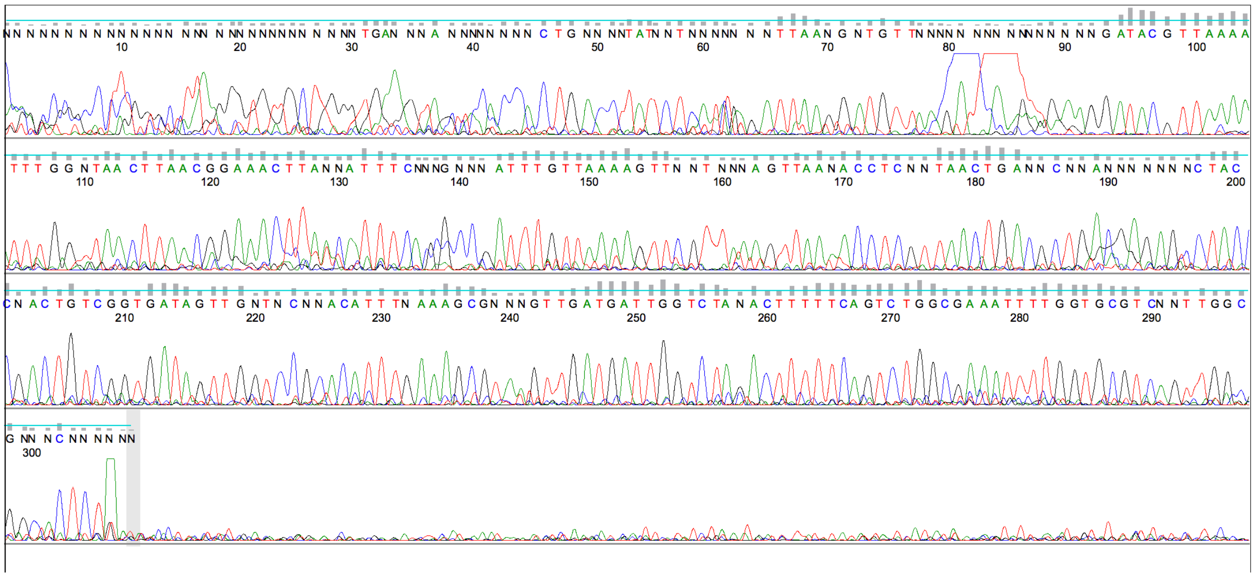

Supplementary Fig. S6. (E) Direct sequencing of the right end of XacN1 DNA. The primer used is GCC ACC ACA GCA GAT AGG ACG ATA CCC GTG, corresponding to XacN1 positions 384,005-384,034 (forward).
